# BRR2a Affects Flowering Time via *FLC* Splicing

**DOI:** 10.1371/journal.pgen.1005924

**Published:** 2016-04-21

**Authors:** Walid Mahrez, Juhyun Shin, Rafael Muñoz-Viana, Duarte D. Figueiredo, Minerva S. Trejo-Arellano, Vivien Exner, Alexey Siretskiy, Wilhelm Gruissem, Claudia Köhler, Lars Hennig

**Affiliations:** 1 Swedish University of Agricultural Sciences, Department of Plant Biology and Linnean Center for Plant Biology, Uppsala, Sweden; 2 ETH Zürich, Department of Biology, Zürich, Switzerland; The University of North Carolina at Chapel Hill, UNITED STATES

## Abstract

Several pathways control time to flowering in *Arabidopsis thaliana* through transcriptional and posttranscriptional gene regulation. In recent years, mRNA processing has gained interest as a critical regulator of flowering time control in plants. However, the molecular mechanisms linking RNA splicing to flowering time are not well understood. In a screen for Arabidopsis early flowering mutants we identified an allele of *BRR2a*. BRR2 proteins are components of the spliceosome and highly conserved in eukaryotes. Arabidopsis BRR2a is ubiquitously expressed in all analyzed tissues and involved in the processing of flowering time gene transcripts, most notably *FLC*. A missense mutation of threonine 895 in BRR2a caused defects in *FLC* splicing and greatly reduced *FLC* transcript levels. Reduced *FLC* expression increased transcription of *FT* and *SOC1* leading to early flowering in both short and long days. Genome-wide experiments established that only a small set of introns was not correctly spliced in the *brr2a* mutant. Compared to control introns, retained introns were often shorter and GC-poor, had low H3K4me1 and CG methylation levels, and were often derived from genes with a high-H3K27me3-low-H3K36me3 signature. We propose that BRR2a is specifically needed for efficient splicing of a subset of introns characterized by a combination of factors including intron size, sequence and chromatin, and that *FLC* is most sensitive to splicing defects.

## Author Summary

Timing of flowering has a great effect on reproductive success and fitness. It is controlled by many external signals and internal states involving a large set of genes. Here we report that the *Arabidopsis thaliana BRR2a* gene is needed for normal flowering. BRR2 proteins are components of the spliceosome and highly conserved in eukaryotes. BRR2a is needed for splicing of a subset of introns, most noticeably in the transcript of the flowering repressor FLC. Reduced *FLC* expression increased transcription of key floral activators, leading to early flowering in both short and long days. Genome-wide experiments established that full BRR2a activity was required only for a small group of introns. We propose that uncompromised BRR2a activity is most important for efficient splicing of a subset of introns of particular size, sequence and chromatin composition, and that *FLC* is most sensitive to splicing defects.

## Introduction

The switch from the vegetative to the reproductive phase is an important developmental transition in flowering plants. The timing of this transition is regulated by several factors, including endogenous and environmental signals. In Arabidopsis, the photoperiod, vernalization and autonomous pathways involved in flowering time control have been investigated in much detail. More recently, additional pathways such as the age or the ambient temperature mediated pathways have been described [1–4]. Most flowering time pathways converge at the activation of a common set of genes that promote flowering and that are known as floral integrators, namely *SUPPRESSOR OF OVEREXPRESSION OF CONSTANS1* (*SOC1*), *FLOWERING LOCUS T* (*FT*) and *LEAFY* (*LFY*), and at the repression of the major flowering repressor *FLOWERING LOCUS C* (*FLC*) [5].

The daily light duration is sensed by the photoperiod pathway. In temperate climates, Arabidopsis and many other species flower earlier in long day (LD) than short day (SD) conditions [5, 6]. In the photoperiod pathway, CONSTANS (CO) activates *FT* expression in leaves. FT protein is a major mobile flowering-inducing signal and moves through the phloem into the shoot apical meristem (SAM) where it changes vegetative meristem identity to flowering [5]. Normal expression of *CO* in long day (LD) photoperiods requires the histone-binding protein MSI1, and partial loss of MSI1 function such as in the partially complemented *msi1* mutant line *msi1-tap1* leads to reduced expression of *CO*, failure of *FT* and *SOC1* activation and to delayed flowering [7, 8]. *FT* and *SOC1* are repressed by FLC in LD and SD [5]. Prolonged cold (vernalisation) inactivates *FLC* expression thus strongly shortening the time to flowering [9]. The autonomous pathway is known to promote flowering independently of environmental signals. Mutants in the autonomous pathway are extremely late flowering due to strong upregulation of *FLC*. The autonomous pathway genes belong to two main subfamilies: (i) chromatin modifiers such as *FLOWERING LOCUS D* (*FLD*) [10], *FVE* [11, 12] and *RELATIVE OF EARLY FLOWERING 6* [13] and (ii) RNA binding proteins (RBP) such as *FCA* [14], *FPA* [15], *FY* [14], *FLOWERING LOCUS K* (*FLK*) [16, 17] and *LUMINIDEPENDENS* (*LD*) [18]. Together, the autonomous pathway genes form a group of partially independently acting genes rather than a classical linear genetic pathway [19].

Transcripts of many plant genes including most of the flowering-related genes contain several introns. Splicing removes the non-coding introns from pre-mRNAs to form mature mRNA (for review see [20, 21]. The spliceosome is a macromolecular complex consisting of five highly conserved small nuclear ribonucleoprotein particles (snRNPs; U1, U2, U4, U5 and U6) and a large number of stabilizing proteins [22]. The splicing reaction can be functionally divided into several steps, including spliceosome assembly, activation, catalysis, and disassembly of the spliceosomal machinery. During the activation step, DExD/H-box RNA helicases are known to play key roles [23–25]. DExD/H-box RNA helicases belong to a large, highly conserved protein family. These proteins play roles in many biological processes related to RNA metabolism, using energy from ATP hydrolysis [26].

RNA processing is much less studied in plants than in animals and yeast. However, during the last decade, the functional role of transcript processing in plants has received some attention (for review see [20, 21]. Several lines of evidence support a connection between RNA processing and flowering time control [27–30]. The proteins identified in pre-mRNA processing were involved in either 3’ end polyadenylation or 5’ end capping. However, little is known about the possible regulatory role of key proteins of the spliceosomal complex in control of flowering.

Here we describe an early flowering allele of Arabidopsis *BRR2a*, which encodes a highly conserved spliceosome protein in eukaryotes. The single missense mutation of threonine at position 895 is associated with an early flowering phenotype. We demonstrate that defects in *FLC* splicing form the mechanism underlying the flowering phenotype. Genome-wide experiments established that full BRR2a activity was required only for a small group of introns. We propose that BRR2a is specifically needed for efficient splicing of a subset of introns characterized by a combination of risk factors in intron size, sequence and chromatin composition, and that *FLC* is most sensitive to splicing defects.

## Results

### The *cäö* mutant causes early flowering

To understand the molecular mechanisms underlying the control of the floral transition by MSI1 [7, 8], a mutant screen for suppressors of the late flowering phenotype of *msi1-tap1* plants was performed, resulting in six mutants with a shortened vegetative phase of *msi1-tap1* [31, 32]. Two suppressor mutants had been reported previously [31, 32]. Here we describe the analysis of one of the remaining uncharacterized suppressor mutants, which was initially called *chrottapösche* (*cäö*) (Swiss German for dandelion) because of its increased leaf serration.

**Fig 1:**
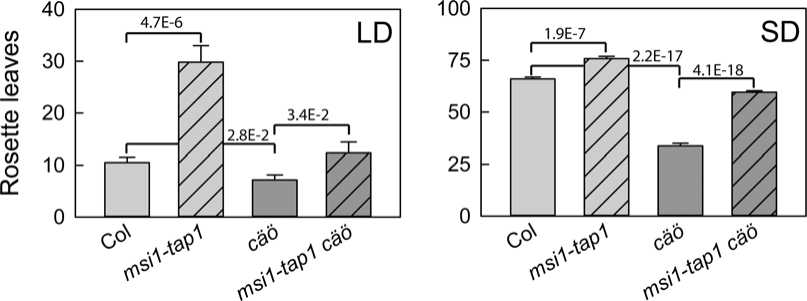
*cäö* is an early flowering mutant in Arabidopsis. Flowering time of wild type (Col), *msi1-tap1*, *cäö* and *cäö msi1-tap1* in number of total rosette leaves at bolting under LD (left) and SD (right). Shown are mean ± SE (n ≥ 14). Numbers in the graph represent p-values of two-sided t-tests.

To test whether the *cäö* early flowering phenotype was independent of the *msi1-tap1* background, *cäö* was backcrossed to Columbia (Col), and flowering time was measured. Under LD and SD conditions, *msi1-tap1* flowered much later than Col, confirming earlier results [7, 8], while *msi1-tap1 cäö* flowered at a similar time to Col (Fig 1). In the Col background, *cäö* flowered earlier than both Col and *msi1-tap1 cäö*, demonstrating that the effect of *cäö* does not require the *msi1-tap1* background. Therefore, *cäö* in the Col background was used in all subsequent experiments.

**Fig 2:**
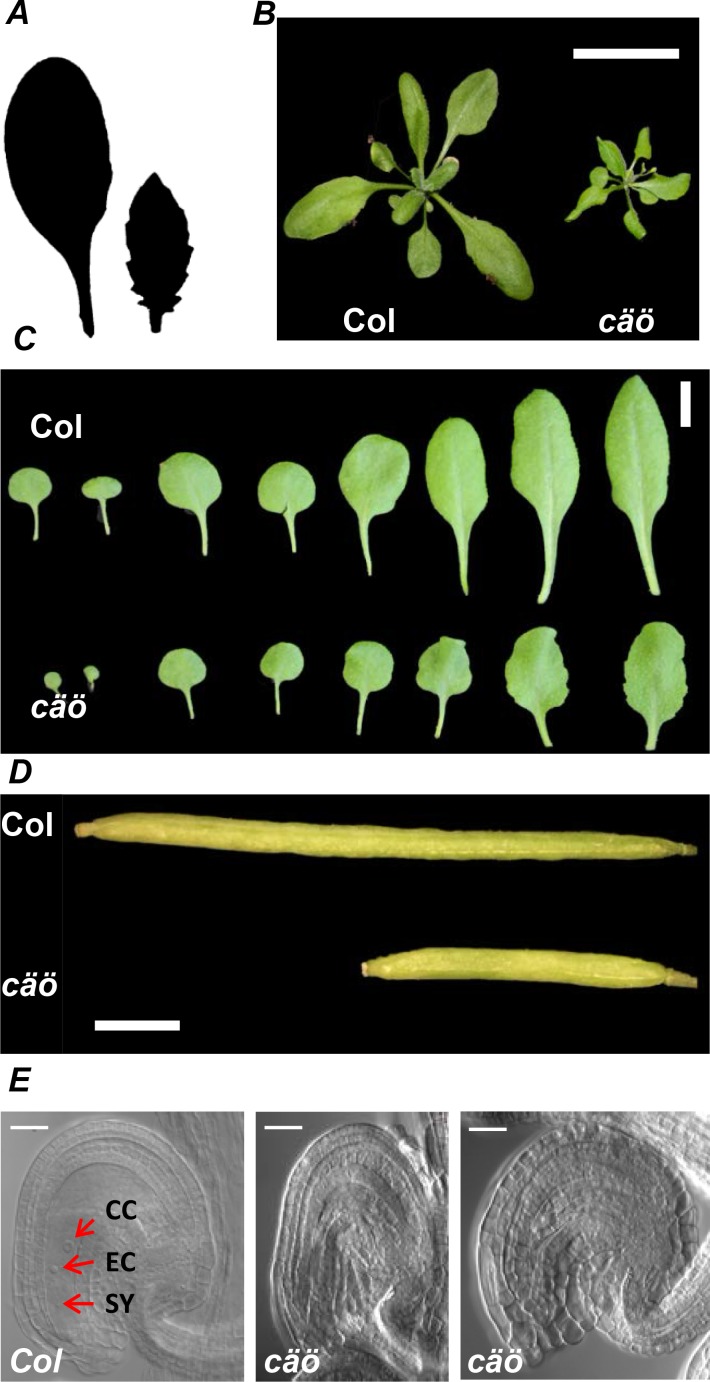
Developmental alterations in *cäö*. (A) Silhouette of the sixth rosette leaf from wild type (Col, left) and *cäö* (right) showing the serrated margin of the *cäö* leaf. Plants were grown for 4 weeks under LD conditions. (B) Rosette morphology of Col and *cäö* plants at time of bolting in LD; scale bar: 5 cm. (C) Leaf morphology of Col and *cäö* in 20 days old plants; scale bar: 1 cm. (D) Reduced silique length in *cäö* mutants. Scale bar: 1 mm (E) Cleared wild type (left panel) and *cäö* ovules with arrested (middle) or absent female gametophytes (right panel). Scale bar: 25 μm. Cells of the egg apparatus are indicated: CC, central cell; EC, egg cell; SY, synergids.

In addition to early flowering, the *cäö* plants had other developmental defects (Fig 2). Leaves of *cäö* were small and serrated contributing to a smaller and more compact rosette (Fig 2A–2C, S1A Fig). Siliques of *cäö* were shorter than WT (Fig 2D), had reduced seed set and contained unfertilized ovules. In about 20% of *cäö* ovules female gametophyte development was delayed or completely absent (Fig 2E, S1B Fig). Defective female gametophyte development had a sporophytic origin because it was mainly observed in homozygous *cäö*^-/-^ plants and only occasionally in heterozygous *cäö*^-/+^ plants (S1B Fig). Together, *CÄÖ* is important not only for normal flowering time but also other developmental programs including formation of female gametophytes.

### *CÄÖ* encodes an ATP-dependent RNA helicase protein

**Fig 3:**
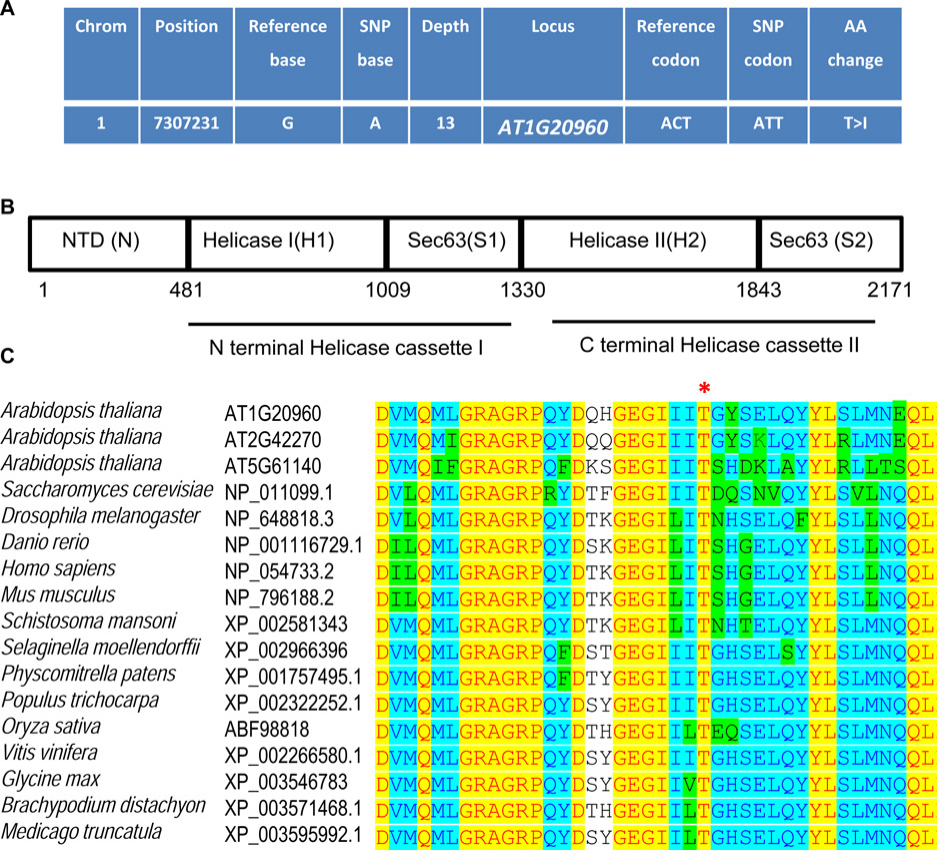
*CÄÖ* encodes the ATP-dependent RNA helicase protein BRR2a. (A) SNP annotations in the identified region with reduced recombination on left arm of chromosome 1. (B) Schematic representation of the protein domain structure of BRR2a. A detailed description of the protein domains can be found in the main text. (C) Threonine 895 is conserved among eukaryotic BRR2 proteins. Sequence alignment of the end of helicase domain 1 in BRR2A proteins from yeast, animals and plants. The asterisk highlights threonine 895, which is altered to an isoleucine in *brr2a*-2. Conserved amino acid residues are highlighted in black. Residues not identical but similar are highlighted in gray.

In order to isolate the causative mutation in *cäö*, a mapping population was established by crossing *cäö* in the Col background with L*er* followed by next generation sequencing of F2 bulk segregants. A candidate region on the left arm of chromosome 1 (S2 Fig) contained only one mutation that was represented in all reads covering this region. The mutation was a G to A transition in the *AT1G20960* gene and caused a T895I missense mutation (Fig 3). This mutation was subsequently confirmed by a specific dCAPS (derived cleaved amplified polymorphic sequences) molecular marker (S3A Fig) and Sanger sequencing (S3B Fig). *AT1G20960*, which was previously identified as *EMBRYONIC LETHAL 1507* (*EMB1507*) [33], encodes an orthologue of yeast Brr2p (Bad response to refrigeration 2 protein) and is also called BRR2a [34]. Yeast Brr2p is a DEAD/DExH box ATP-dependent RNA helicase with a unique N-terminal domain and two consecutive helicase cassettes (with a DExD/H domain and helicase superfamily C-terminal domain), each followed by a Sec63 domain (Fig 3B) [35, 36]. Yeast and animal BRR2 proteins are integral components of the U5 small nuclear ribonucleoprotein (snRNP) and are essential for splicing through their contribution to the recruitment and activation of spliceosome complex components [24, 37]. Because Brr2 is the original name given to these proteins, we refer to the mutant protein as BRR2a-T895I and to *cäö* as *brr2a*-2. The mutated threonine 895 is located at the end of the first helicase domain and highly conserved in Brr2 proteins (Fig 3C), suggesting that the T895I mutation interferes with BRR2a function.

To confirm that the early flowering phenotype is caused by the disruption of *BRR2a*, an allelism test was performed. Heterozygous *emb1507*-4 null mutant plants were used to pollinate homozygous *brr2a*-2 plants. All F1 plants with the *emb1507*-4 allele but none of the plants without it displayed the *brr2a*-2 phenotype, establishing that the mutant BRR2a protein caused the *cäö* mutant phenotype. Considering the recessive nature of the *brr2a*-2 mutant, these data suggest that *brr2a*-2 is a hypomorphic rather than a neomorphic allele and that the *cäö* phenotype is caused by reduced activity of BRR2a.

### BRR2a is a highly conserved protein

BRR2 sequences from different eukaryotes including yeast, metazoa, protozoa and plants were aligned and a phylogenetic tree was constructed (S4 Fig). Arabidopsis *BRR2a* has two paralogues, *BRR2b* (*At2g42270*) and *BRR2c* (*At5g61140*). *BRR2a* and *BRR2b* result from a recent gene duplication and are part of a clade with members in all green plants. In contrast, BRR2c belongs to a minor clade with only one protein from the fern *Selaginella* and one yeast protein. Furthermore, BRR2a shares 82% identity and 91% similarity with BRR2b but only 40% identity and 59% similarity with BRR2c (S1 Table). Although BRR2a and BRR2b are conserved, the obvious phenotype of the *brr2a* mutant indicates that the genes do not have redundant functions.

Data in the Arabidopsis eFP Browser [38] show that *BRR2* genes are expressed in most tissues at variable levels (S5 Fig), with *BRR2a* having the highest transcript levels. *BRR2c* was expressed considerably less than *BRR2a*, and *BRR2b* was expressed lowest of the three *BRR2* homologues in most tissues. It is likely that the high expression of *BRR2a* accounts for the strong phenotypes of *brr2a* mutants.

### *FLC* expression levels are altered in *brr2a*-2

**Fig 4:**
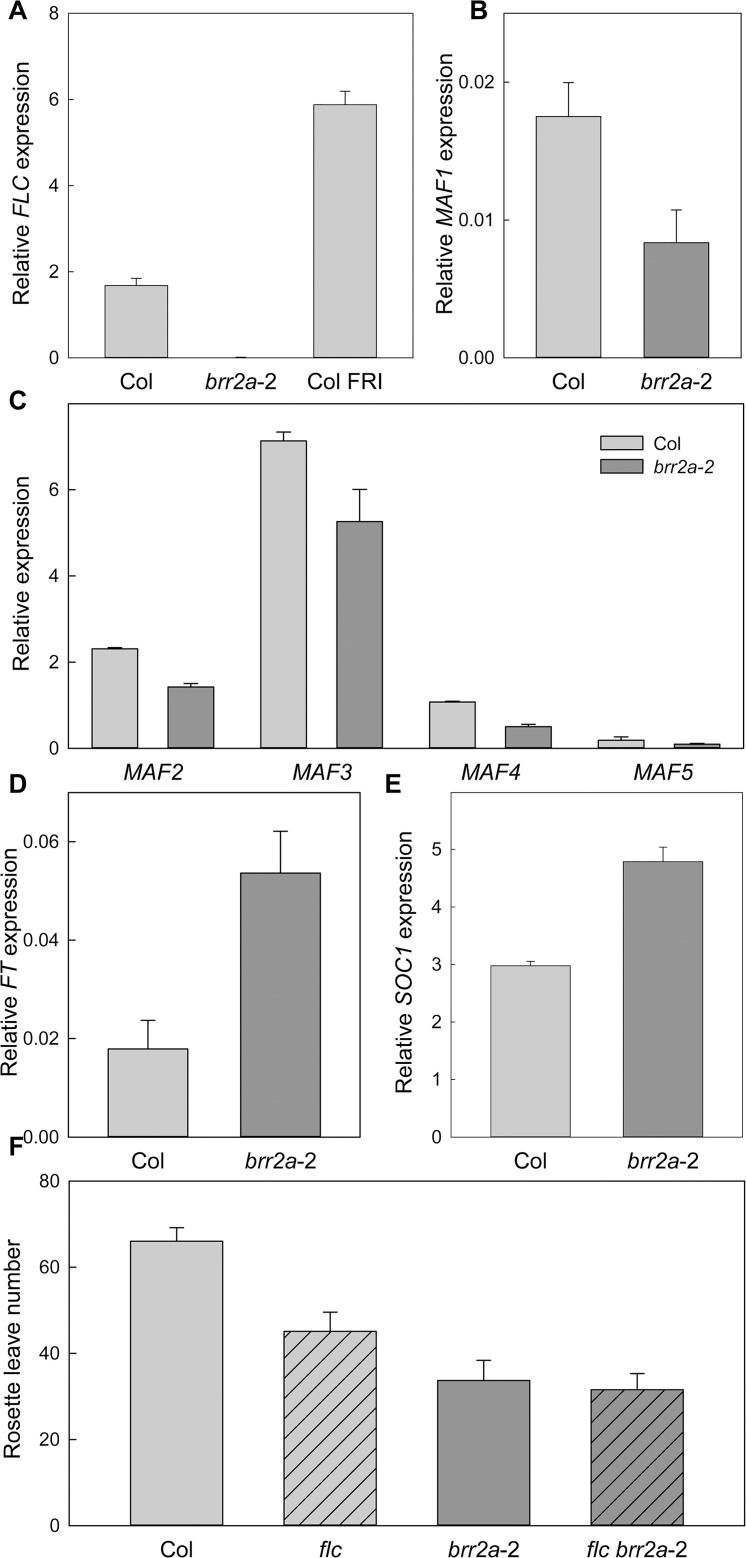
*FLC* transcript levels are altered in *brr2a*-2. (A) Transcript levels of the flowering repressor gene *FLC* in Col, *brr2a*-2 and *FRI*. (B-C) Transcript levels of the *MAF1* (B) and *MAF2*—*MAF5* (C) in Col and *brr2a*-2. (D-E) Transcript levels of the floral integrators *FT* (D) and *SOC1* (E) in Col and *brr2a*-2. Quantitative RT-PCR in (A-E) was performed using RNA from 15 day-old seedlings grown under SD conditions, at *zeitgeber* time (ZT) = 7. Relative expression to *PP2a* is shown as mean ± SE (n = 3). (F) Genetic interaction of *brr2a*-2 and *flc* for flowering time control. Flowering time of Col, *flc*, *brr2a*-2 and *flc brr2a*-2 in number of total rosette leaves under SD. Shown are mean ± SE (n ≥ 14).

FLC is a MADS-box DNA binding protein and a major repressor of flowering time in Arabidopsis [39, 40]. Many early flowering Arabidopsis mutants have reduced *FLC* expression whereas many late flowering mutants have increased *FLC* expression. We tested for possible changes of *FLC* transcript levels in 15 days old seedlings grown under SD conditions. Col plants containing an active *FRIGIDA* (*FRI*) allele, which express high levels of *FLC*, were included as control. As reported before, the expression levels of *FLC* were much higher in *FRI* than in Col [41]. In contrast, *FLC* levels were strongly (>95%) reduced in *brr2a*-2 (Fig 4). The *MADS AFFECTING FLOWERING* (*MAF*) genes are homologues of *FLC* and are often similarly regulated [42, 43]. In *brr2a*-2, expression levels of *MAF1* and *MAF4* mRNAs were about 50% reduced and *MAF2*, *MAF3* and *MAF5* mRNA levels were also lower than in WT (Fig 4B and 4C). Together, the results are consistent with the observed *cäö* early flowering phenotype and the effect of *FLC* on flowering time control [39].

### *FT* and *SOC1* expression is increased in *brr2a*-2

*FT* and *SOC1* promote flowering and both are repressed by FLC. We therefore tested if early flowering of *brr2a*-2 was associated with increased *FT* and *SOC1* expression. The expression of *FT* was nearly three times higher in *brr2a*-2 than in Col (Fig 4D) and *SOC1* had significantly increased expression as well (Fig 4E). These results are consistent with the decreased *FLC* levels in *brr2a*-2 and indicates that early flowering is caused by increased expression of *FT* and *SOC1* that have been released from the repression by FLC.

We tested genetically whether *brr2a*-2 accelerates flowering via *FLC* by measuring flowering time of the double *brr2a*-2 *flc* mutant (Fig 4F). Consistent with earlier reports, *flc* flowered earlier than Col. The *brr2a*-2 mutant flowered even earlier than *flc*, possibly because of reduced expression of other floral repressors such as the *MAF* genes. Importantly, the *brr2a*-2 *flc* double mutant did not show further acceleration of flowering revealing complete epistasis of *BRR2a* and *FLC*, which is fully consistent with the reduced *FLC* expression as the major cause of accelerated flowering in *brr2a*-2.

### Transcript processing of *FLC* is altered in *brr2a*-2

**Fig 5:**
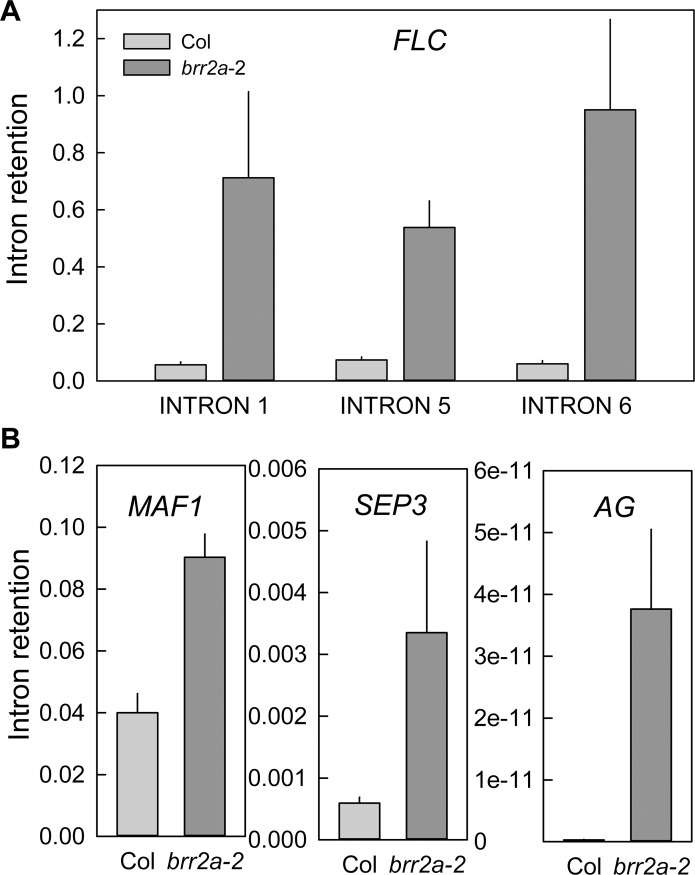
*FLC* splicing efficiency is reduced in *brr2a*-2. Intron retention was calculated as the ratio of unspliced to total (spliced + unspliced) transcripts for three representative *FLC* introns (A) and for intron 1 in *MAF1* and *SEP3*, and intron 2 in *AG* (B) in both Col and *brr2a*-2. For *FLC* and *MAF1*, RNA was extracted from 15 day-old seedlings grown under SD conditions at ZT = 7. For *SEP3* and *AG*, RNA was extracted from inflorescences of LD-grown plants. Results were normalized to *PP2a*; shown are mean ± SE (n = 3). Note the different scales used for each gene showing that that majority of *MAF1*, *SEP3* and *AG* transcripts are correctly spliced even in the *brr2a*-2 mutant.

The reduced *FLC* transcript levels did not correlate with altered transcript levels of major *FLC* activators or repressors (S6 Fig). Splicing of *COOLAIR*, an antisense transcript covering the *FLC* locus [44], contributes to repression of *FLC* transcription and depends on a homolog of the yeast spliceosomal PRP8 protein [30]. *COOLAIR* splicing is strongly disrupted in *brr2a*-2 (S7 Fig) and reduced levels of *COOLAIR* and *FLC* transcripts are consistent with correlated *FLC* and *COOLAIR* production [44]. In contrast to a *prp8* mutant in which defective *COOLAIR* splicing increased *FLC* transcription [30], defective *COOLAIR* splicing in *brr2a*-2 did not increase *FLC* transcript levels. Therefore we tested whether the reduced *FLC* transcript levels in *brr2a*-2 were due to defects in *FLC* transcript splicing. Intron retention (IR) was tested by qPCR for randomly selected *FLC* introns 1, 5 and 6. For all three tested *FLC* introns, IR was about 8-fold higher in *brr2a*-2 than in Col (Fig 5A). These results suggest that the BRR2a-T895I mutation resulted in an unproductive splicing complex that caused IR and reduced accumulation of correctly spliced *FLC* in *brr2a*-2. Incorrectly spliced transcripts are subject to nonsense-mediated decay (NMD) mRNA quality control and have a much higher turnover rate than correctly spliced transcripts [45], which could explain the reduced *FLC* transcript levels.

### BRR2a-T895I affects the splicing efficiency of a small group of introns

The splicing defects of the *FLC* transcript can explain the early flowering phenotype but it was possible that in *brr2a*-2 transcripts of other genes were affected as well. We used PCR assays to test IR in transcripts of the three MADS-box genes *MAF1*, *AG* and *SEP3*, which have a similar intron-exon structure as *FLC*. For each of these genes, retention of the intron corresponding to *FLC* intron 1, which showed strong retention in *brr2a*-2, was tested. Although IR was increased in the transcripts of all three genes (Fig 5B) it never exceeded 10% and was thus much less than for *FLC* of which 90% of the transcripts retained intron sequences, suggesting that not all transcripts depend to the same extent on functional BRR2a for correct processing.

To investigate other potential splicing defects in *brr2a*-2 we performed an RNA-seq experiment. For each of three wild-type and three mutant libraries between 23 and 40 million reads where generated of which 83–92% could be mapped to the Arabidopsis TAIR10 reference genome. About 90% of the mapped reads were from non-overlapping, annotated genes (S2 Table) and 4% of the mapped reads corresponded to intron sequences. Analysis of differentially expressed genes (DEG) identified 279 genes with increased and 103 genes with decreased transcript levels (S3 and S4 Tables). The gene with the strongest transcript reduction was *FLC* (6.6 fold, p = 2.1E-15) and consistent with the early flowering phenotype, *SOC1* expression was significantly upregulated (3.1 fold, p = 7.7E-10). In addition, the transcript level of the *BRR2a* homolog *BRR2b* was significantly increased (2.8 fold, p = 1.6E-11). Similarly, the gene encoding the predicted splicing factor AtPRP8b, which is thought to function together with BRR2 [34], was upregulated in *brr2a*-2 (3.2 fold, p = 4.3E-08). Upregulation of *BRR2b* and *AtPRP8b* expression could be caused by an autoregulatory loop responding to a reduced function of the BRR2a-T895I mutant protein. It is possible that the upregulation of *BRR2b* and *AtPRP8b* distort stoichiometry in the splicosome and enhance the defects in *brr2a*-2. Among the upregulated DEGs, four gene ontology (GO) categories were significantly overrepresented (p<0.05, S5 Table). The GO categories "response to UV-B" and "response to salicylic acid stimulus" are consistent with earlier reports of connections between splicing and stress responsiveness [21]. Also the categories "regulation of transcription, DNA-dependent" and “response to karrikin” were enriched among the upregulated genes. Only one GO category ("proteolysis") was significantly enriched (p = 0.02) among the downregulated genes (S6 Table). Next, the transcriptome data were searched for misexpression of genes involved in leaf development. Of 103 genes from GO category GO:0009965 (leaf development) for which transcripts were detected, three were significantly stronger expressed in *brr2a*-2 than in wild type: *TEOSINTE BRANCHED 1*, *CYCLOIDEA*, *AND PCF FAMILY 13* (*TCP13*), *KIPRELATED PROTEIN 6* (*KRP6*) and *KRP1* (S8A Fig). Two leaf development genes were less expressed in *brr2a*-2 than in wildtype: *ASYMMETRIC LEAVES 1* (*AS1*) and *TCP24*. Reduced expression of both *TCP24* and *AS1* is consistent with earlier reports that TCP24 is an activator of *AS1* [46]. Increased expression of *KRP6* or *KRP1* causes reduced leaf size and increased serration [47, 48] and is consistent with the leaf phenotype of *brr2a*-2 plants.

**Fig 6:**
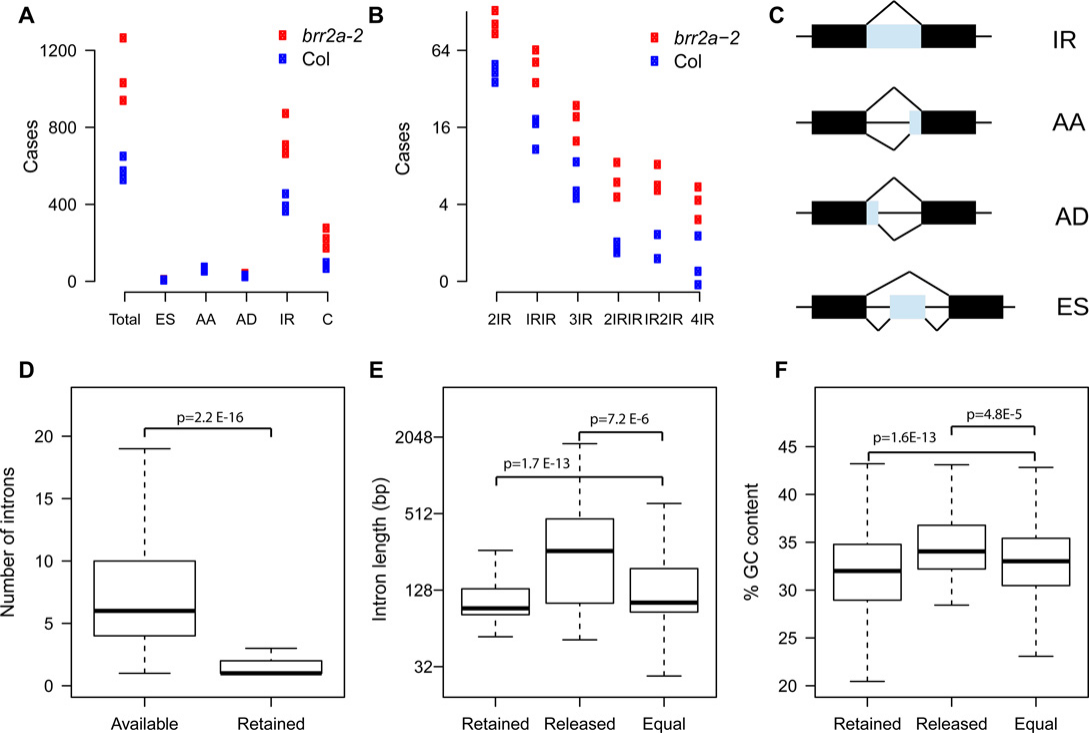
Intron retention in *brr2a*-2. (A) Number of alternative splicing events (AS) based on three biological replicates each of Col and *brr2a*-2. ES, exon skipping; AA, alternative acceptor site; AD, alternative donor site; IR, intron retention; C, complex, a combination of one or several of ES, AA, AD or IR. The increased number of AS events in *brr2-a* is mainly due to increased intron retention. (B) Number of complex alternative splicing events. 2IR, two intron retention events in the same transcript; IRIR, either one of two different introns is retained; 3IR, three intron retention events in the same transcript; 2IRIR, either a pair of introns or a more 3' located single intron is retained; IR2IR, either a single intron or a more 3' located pair of introns is retained; 4IR, four intron retention events in the same transcript. For (A) and (B), AS events were quantified from RNA-seq data using ASTALAVISTA [49]. The complex AS events observed in *brr2a*-2 are mainly combinations of IR events. (C) Schematic representation of basic AS events. (D) Total intron number per transcript with detected IR (left) and number of retained introns per transcript with detected IR (right). Whereas transcripts with detected IR have on average 6 transcripts, only one or two of those are retained, suggesting that IR is mostly an intron and not a transcript property. (E) Length of introns with increased retention in *brr2a*-2 (“retained”), with decreased retention (“released”) and with no change in retention (“equal”). Introns with increased retention are often shorter whereas introns with decreased retention are often longer than unaffected introns. (F) GC content of introns with increased retention in *brr2a*-2 (“retained”), with decreased retention (“released”) and with no change in retention (“equal”). Introns with increased retention have often lower GC content whereas introns with decreased retention have often higher GC content than unaffected introns. P-values are from one-sided t-tests.

The ASTALAVISTA program suite [49] was used to collate alternative splicing (AS) events in the six libraries (Fig 6). Relative frequencies of AS events in WT were similar to those reported earlier [50]. In *brr2a*-2, almost twice as many AS events were detected than in wild type (Fig 6A). This increased AS frequency was caused mainly by IR and partly by more complex events such as double intron retention (Fig 6A–6C). Exon skipping (ES) as well as use of alternative acceptors or donors did not differ between wild type and mutant. Therefore, we focused on IR events and used DESeq2 [51] with numbers of intron-derived reads to detect differentially retained introns (DRIs). There were 914 DRIs with significantly increased but only 74 with decreased retention (S7 and S8 Tables). Thus, the BRR2a-T895I mutation causes primarily increased intron retention. The low number of retained introns (1.2% of 74’581 introns with available read counts or 0.8% of 117’458 analyzed introns) indicates that only a specific subset of introns is strongly affected in *brr2a*-2. RT-PCR assays using independent RNA samples confirmed increased IR in *brr2a*-2 for 10 out of 10 tested genes, suggesting a low false positive rate of detected DRIs (S9 Fig). Of the leaf development genes with altered expression in *brr2a*-2 (S8A Fig) only intron 1 of *TCP13* was significantly differentially retained (p = 1.1E-02; S8B Fig). The weaker retention of the other intron of *TCP13* was not significant (p>0.05). The differentially expressed leaf development genes *KRP1*, *KRP6*, *TRCP24* and *AS1* all contain introns but did not show signs of increased IR in *brr2a*-2 plants (S8B Fig). Thus, it is possible that the leaf development phenotype of *brr2a*-2 *is* related to splicing defects in the *TCP13* transcription factor.

It was possible that certain genes depend critically on BRR2a for splicing of several introns. However, although the genes with DRIs contain on average six introns, for most of them only a single intron was significantly retained (Fig 6D). Thus, intron retention in *brr2a*-2 appears to be intron-specific rather than a gene or transcript property.

It was possible that DRIs are characterized by specific sequence signatures. We used the R package motifRG to test whether DRIs contained enriched motifs using the sequences of unchanged introns as background. However, there were no sequence motifs enriched in DRIs over unchanged introns. Similarly, splice site sequences did not differ between DRIs and unchanged introns (S10 Fig). Next, we tested whether DRIs differ in length from unchanged introns. Less efficiently spliced DRIs were significantly shorter than unchanged introns (mean of 140 bp vs. 173 bp; p = 1.7E-13, one-sided t-test) (Fig 6E). Conversely, more efficiently spliced DRIs where significantly longer than unchanged introns (mean of 360 bp vs. 173 bp; p = 7.2E-6, one-sided t-test). In addition to length, DRIs differed also in GC content from unaffected introns (Fig 6F). Less efficiently spliced DRIs had a significantly lower GC content than unaffected introns (31.8% vs. 32.9%; p = 1.6E-13, one-sided t-test); and more efficiently spliced DRIs had a significantly higher GC content than unaffected introns (35% vs. 32.9%; p = 4.8E-5, one-sided t-test). We also tested an effect of intron folding using but stability of the most likely secondary structure did not differ between retained and spliced introns (see Materials and Methods for details). Thus, the BRR2a-T895I mutation appears to reduce splicing efficiency preferentially of short and GC-poor introns and shifts it to longer, GC-rich introns.

**Fig 7:**
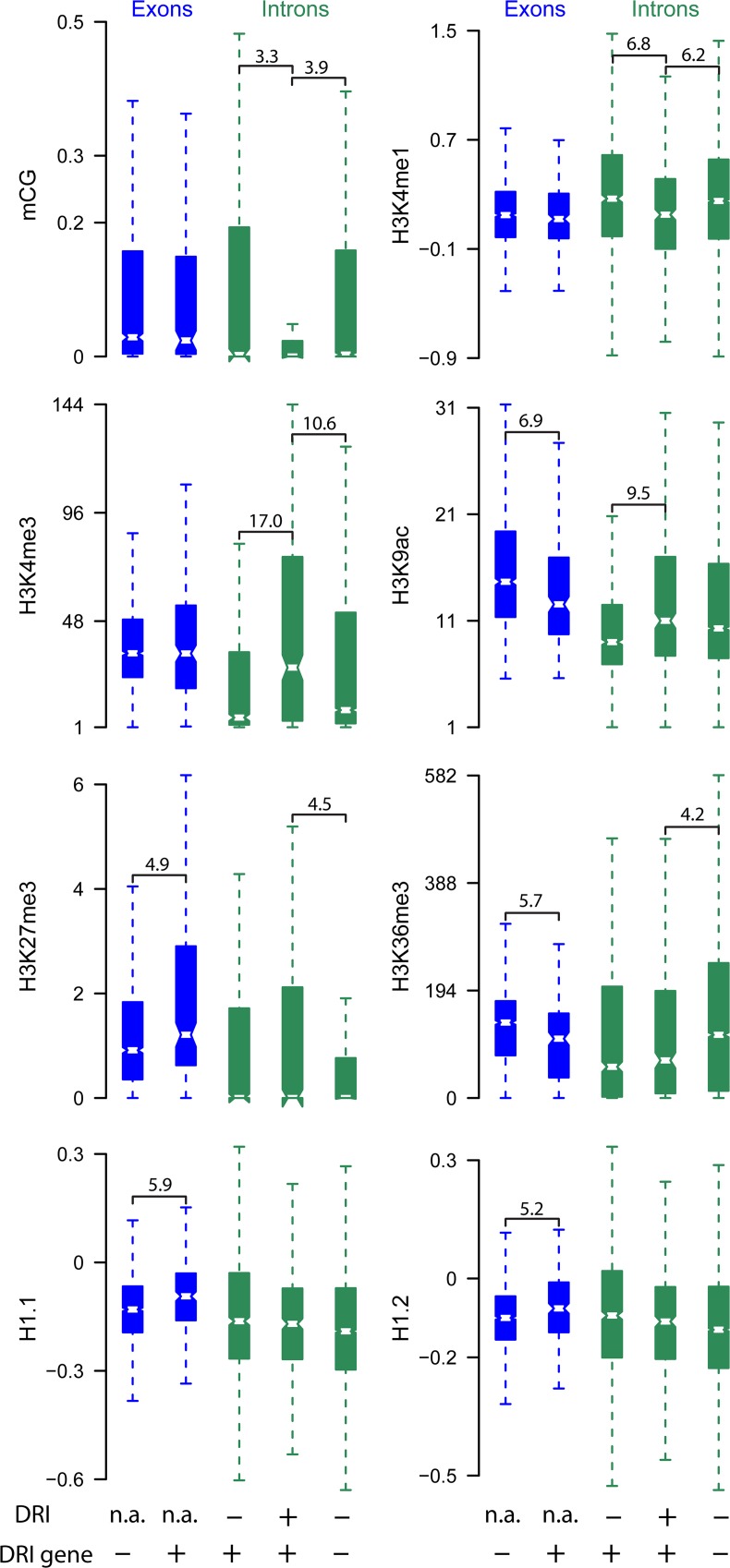
Intron retention in *brr2a*-2 is associated with specific chromatin properties. Box plots show averaged genome-wide bisulfite sequencing and ChIP signals for exons (blue) and introns (green). DRI, differentially retained introns in *brr2a*-2. DRI gene, genes containing at least one differentially retained intron. Thus, the boxes in each plot represent, from left to right, (i) exons from genes that have no DRIs, (ii) exons from genes that have at least one DRI, (iii) normally spliced introns in genes that have at least one DRI, (iv) DRIs, and (v) introns from genes that have no DRIs. Control genes without DRIs were selected to have the same median expression as the DRI genes. Significant differences are indicated; numbers are–log_10_ of p-values from Wilcoxon signed-rank tests.

Because chromatin can affect splicing [52], we tested whether chromatin on DRIs differs from chromatin on other introns. To exclude confounding effects by transcription strength, DRIs were compared to two different control sets: (i) Non-differentially retained introns from the genes that have at least one DRI, and (ii) introns from a set of genes that do not contain DRIs and that have a similar median expression as the genes with DRIs. DRIs did not differ significantly from control introns regarding CHG methylation, CHH methylation, H3 density and H3K9me2 (S11 Fig). In contrast, DRIs had less H3K4me1 and CG methylation (mCG), and more H3K4me3 than control introns (Fig 7). H3K9ac was higher on DRIs than on the non-DRI introns in the same genes but similar to the level on introns of control genes. H3K27me3 and H3K36me3 did not differ between DRIs and non-DRI introns in the same genes but were higher and lower, respectively, on exons and introns of genes with DRIs than on control genes. In addition, exons on genes with DRIs had more H1.1, H1.2, and H3K27me3 and less H3K9ac than exons on control genes. Thus, BRR2a function is most important on genes generally rich in H3K27me3 and H1, and low in H3K36me3 and H3K9ac. It is also most important for splicing of introns with low mCG and H3K4me1, and high H3K4me3. This shows that local chromatin properties can affect the outcome of a mutation in a spliceosomal subunit.

## Discussion

Yeast and human BRR2 proteins are evolutionary highly conserved spliceosome proteins of about 200 kDa [53, 54]. They belong to the DEAD/DExH-box family of ATP-dependent RNA helicases with two putative RNA helicase domains, each with a highly conserved ATPase motif, followed by a SEC63 domain. Their ATPase and helicase activities are involved in rearrangements necessary for spliceosome activation through the unwinding of U4/U6 snRNP [24]. After the unwinding, BRR2 remains stably associated with the catalytic core of the spliceosome [55] and eventually promotes spliceosome disassembly by unwinding U2/U6 [56]. BRR2 also functions in promoting conformational rearrangements in the spliceosome during the first-to-second-step transition, which aid 3’ splice site positioning and formation of the second-step catalytic center [57]. BRR2 helicase activities are highly regulated to ensure the correct timing of spliceosome activation or disassembly [58]. Regulators of BRR2 functions include Prp8 [59, 60] and the Snu114 GTPase [56].

Here, we identified a new allele of BRR2a containing a T895I mutation, which enabled us to demonstrate that Arabidopsis BRR2a functions in intron splicing. In BRR2a, threonine 895 and its neighboring amino acid sequences are highly conserved and located near the ATPase domain within the first conserved helicase I motif. A crystal structure and structural models for different yeast and human BRR2 helicase regions show a possible reorganization and pairing of these domains during the splicing process [61]. Furthermore, mutagenesis studies in the amino-terminal helicase domain revealed the role of this domain in splicing efficiency [23, 62]. Together, the T895I mutation in *brr2a*-2 likely results in a partial loss of BRR2a function or impairs interaction of BRR2a with other proteins in the spliceosome. Plausible interactors are PRP8 and GAMETOPHYTE FACTOR 1 (GFA-1) [34], which are Arabidopsis homologues of the Brr2p regulatory yeast proteins Prp8 and Snu114 [63], respectively. Indeed, GFA1 interacts with the carboxy-terminal domain of BRR2a and with PRP8a in yeast two-hybrid assays [34] and BRR2a and PRP8a copurify [64]. However, it is not known whether mutation of T895 in the amino-terminal domain affects this interaction. Other BRR2a candidate interactors are homologues of the serine/threonine protein kinase Prp4 and its substrates such as Prp1. These proteins interact biochemically with fission yeast Brr2p. Future experiments will establish whether the T895I mutation affects BRR2a’s ability to interact with other proteins. An alternative explanation for reduced function of BRR2a-T895I could be that the T895I mutation weakens BRR2a interactions with U4/U6, U5 or pre-mRNAs. This appears plausible because *in vivo* UV cross-linking and RNA sequencing have established that budding yeast Brr2p binds not only to the U4/U6 and U5 snRNA but also to pre-mRNAs [57]. It is likely that the polar T895 is surface-exposed, and if a change at this residue reduces BRR2a-RNA interactions, this could also reduce the activity of BRR2a-T895I in the splicing reaction.

In Arabidopsis, the BRR2a-T895I mutation results in early flowering and altered leaf development. Splicing defects in the transcripts of the *TCP13* transcription factor and altered transcript levels of *TCP13*, *TCP24*, *AS1*, *KRP1* and *KRP6* are consistent with the leaf phenotype of *brr2a*-2 plants. KRPs repress cell proliferation by inhibiting cyclin-dependent kinases [65] / Sablowski, 2014 #13362]. Increased expression of *KRP6* or *KRP1* causes reduced leaf size and increased serration [47, 48] consistent with the concomitant upregulation of these genes and altered leaf size and shape in *brr2a*-2. TCPs are major transcriptional regulators that control cell proliferation in leaves (for reviews see [66, 67]). TCPs related to TCP13 (CIN-like TCPs) function highly redudnantly [68]; they promote the arrest of cell division, and overactivation can strongly reduce leaf size and increase serration. Because the CIN-like TCP13 homolog TCP4 is a direct activator or *KIP1* [69], it is possible that increased expression of *TCP13* causes *KRP* upregulation, leading to premature arrest of cell proliferation and reduced leaf size in *brr2a*-2. Retention of *TCP13* intron 1 does not alter transcript coding potential as this intron 1 is located in the 5’ untranslated region; it could, however, affect translation efficiency.

FLC is a major repressor of flowering that functions by repressing *FT* and *SOC1* [39, 40, 70, 71]. Expression and genetic data support the model that reduced *FLC* transcript levels in *brr2a*-2 allow untimely activation of *FT* and *SOC1*, which together cause accelerated flowering. No strong changes in the expression of known regulators of *FLC* were found in *brr2a*-2 but strong defects in *FLC* splicing efficiency were evident from intron retention. Whereas the proportion of intron-containing *FLC* transcripts was increased, total amounts of *FLC* transcripts was decreased in *brr2a*-2. This is similar to the *pep-4* mutant [72] and possibly a consequence of RNA quality surveillance such as NMD, a co-transcriptional quality control pathway that degrades aberrant transcripts [45]. In addition to the NMD pathway, other RNA quality pathways operate in the nucleus and degrade transcripts with delayed processing independent of the presence of stop codons [73]. Although a functional correlation between transcription and transcript processing was previously reported in yeast and mammals [74], it remains unknown to what extent RNA quality pathways can feed back to repress transcription.

Complete loss of Arabidopsis BRR2a function is embryo lethal [33], and the phenotype of the *cäö* mutant suggests that *brr2a*-2 is a hypomorphic allele and that BRR2a-T895I has reduced function. The pleiotropic morphological defects of *brr2a*-2 plants revealed that full BRR2a function is needed in flowering control and also other developmental programs. The developmental role of BRR2a is consistent with previous reports of requirements for spliceosome components in animal and plants development [34, 75–79]. In cases of embryo lethality hypomorphic alleles such as *brr2a*-2 constitute unique and powerful resources to analyse gene functions during postembryonic life. The restricted phenotypic changes of *brr2a*-2 plants are also consistent with the result that splicing of only a group of introns was affected. Thus, introns differ in their dependency on BRR2a activity, and *FLC* is particularly sensitive to the BRR2a-T895I mutation. Our discovery of the *cäö* mutant has revealed that mutations in different spliceosome proteins can affect distinct sets of introns. We have shown that *FLC* splicing is greatly reduced in *brr2a*-2 while a recent study showed that *FLC* splicing was normal in a *prp8* mutant [30].

Genome-wide sequencing data indicate that retained introns often came from genes with a high-H3K27me3-low-H3K36me3 signature. Retained introns differed also in their local chromatin composition and had more H3K4me3, less H3K4me1 and less mCG than control introns. In addition, splicing efficiency in *brr2a*-2 plants was often shifted from short, GC-poor to long, GC-rich introns. However, the case of *FLC* shows that even long introns can have splicing defects in *brr2a*-2. In addition, not all short introns were retained in *brr2a*-2 consistent with a combined effect of intron length, chromatin features and maybe other properties. This notion is supported by the observation that *FLC*, which contains a retained long intron, has the high-H3K27me3-low-H3K36me3 signature. Similar to other retained introns, intron 1 of *FLC* has low H3K4me1. Only 0.3%, 31.6% and 14.1% of all introns have higher H3K27me3, lower H3K36me3 or lower H3K4me1, respectively, than intron 1 of *FLC*. In contrast, H3K4me3 and mCG on *FLC* intron 1 were close to the average of all introns.

Our finding that retained introns have particular chromatin signatures is consistent with recent reports about effects of chromatin on splicing in animals (for review see [52, 80]. Although the underlying mechanisms are poorly understood, two main models have been developed: (i) In the kinetic coupling model, local chromatin affects the rate of RNA Polymerase II (Pol II) elongation. Slow elongation or pausing favors weak splice sites while fast elongation misses weak sites and favors downstream strong sites. (ii) In the recruitment coupling model, chromatin-binding adaptor proteins recruit specific splicing regulators to define splicing outcome [52, 80]. Alternative splicing of the human fibroblast growth factor receptor 2 (*FGFR2*), for instance, is mediated by H3K36me3-based recruitment of the polypyrimidine tract–binding protein splicing factor [81]. Notably, one of the alternative spliced states of human *FGFR2* shares the high-H3K27me3-low-H3K36me3 chromatin signature of Arabidopsis genes with IR in *brr2a*-2 [81]. More recently, alternative splicing of *FGFR2* was found to be affected by a long non-coding antisense RNA that recruits Polycomb-group proteins and the H3K36 histone demethylase KDM2 to establish a high-H3K27me3-low-H3K36me3 chromatin signature [82]. We note that also at Arabidopsis *FLC*, a long antisense RNA affects H3K36me3 to establish a high-H3K27me3-low-H3K36me3 chromatin signature [83]. Future work needs to address whether regulation of *FLC* and *FGFR2* splicing share a common mechanistic basis. In addition to histone modifications, also DNA methylation is associated with splicing [84]. In mammals, introns often have lower mCG than exons, and recruitment of CCCTC-binding factor and methyl-CpG binding protein 2 to mCG can affect splicing by modulating Pol II elongation rates [84]. In maize, it has been proposed that CHG methylation at splice acceptor sites may inhibit RNA splicing [85] and loss of DNA methylation at a splice acceptor site in the oil palm *DEFICIENS* gene was associated with splicing defects in somaclonal variation [86]. It is not known whether mCG affects splicing in Arabidopsis but our results suggest a mechanistic link between mCG and IR.

Despite the similarities between chromatin features found related to splicing outcomes, ES is the most prevalent alternative splicing event in mammals and IR is most prevalent in plants. Therefore, more work is needed to establish whether the mechanisms that couple local chromatin properties to splicing are shared or differ between animals and plants.

In summary, our suppressor mutant screen for accelerated flowering led to the discovery of the early flowering *brr2a*-2 mutant. Our data suggest a model in which BRR2a functions in the spliceosome with the T895I missense mutation leading to reduced splicing efficiency for transcripts of a selected group of genes, most importantly *FLC*. Reduced *FLC* expression allows unscheduled transcription of *FT* and *SOC1* to accelerate flowering. Together, our work establishes correct splicing as an important mechanism for flowering time control and uncovers a complex relation between chromatin features and splicing outcomes in Arabidopsis.

## Materials and Methods

### Plant material and growth conditions

The *Arabidopsis thaliana* wild-type and T-DNA insertion lines were in the Columbia-0 (Col) background. *FRI* in Col [87], *msi1-tap1* and *flc*-6 [7] were described before; *emb1507*-4 (NASC ID: N16092) was obtained from the Nottingham Arabidopsis Seed Stock Centre. The EMS-mutated allele *cäö* in the *msi1-tap1* background was isolated in a mutant screen that was described before [31]. For further characterization, *cäö* in the *msi1-tap1* background was backcrossed into Col. Seeds were sown on 0.5× basal salts Murashige and Skoog (MS) medium (Duchefa, Haarlem, The Netherlands), stratified at 4°C for 2–3 day, and allowed to germinate in growth chambers at 20°C for 10 days under LD (16 h light) or SD (8 h light) photoperiods. Plantlets were planted in soil and grown in growth chambers under the same conditions.

Flower buds were emasculated at anthesis and the non-pollinated pistils were collected 2–4 days after emasculation. The samples were fixed with ethanol-acetic acid (9:1), washed for 10 min in 90% ethanol, 10 min in 70% ethanol and cleared over-night in a chloralhydrate solution (66.7% chloralhydrate (m/m), 8.3% glycerol (m/m)). Ovules were observed under differential interference contrast (DIC) optics using a Zeiss Axioplan microscope (Zeiss, Jena, Germany). Images were recorded using DFC295 Leica camera (Leica, Wetzlar, Germany).

### Mapping by Illumina deep-sequencing

A mapping population was established by crossing *cäö* with the polymorphic ecotype L*er*, and total genomic DNA was extracted from 150 F2 plants presenting the mutant phenotype using the Nucleon Phytopure genomic DNA extraction Kit (Amersham Bioscience, Uppsala, Sweden). After library preparation using standard Illumina protocols, the DNA was loaded onto an Illumina Genome sequencer GA IIx and run for 36 cycles. The obtained short reads were mapped against the TAIR10 release of the Arabidopsis genome using Bowtie 2 [88].

Genome-wide SNP positions and pileup information were then collected and filtered as recommended in the Next-generation EMS mutation mapping software [89].

dCAPS primers were designed using dCAPS Finder 2.0 [90] (S9 Table). The amplified fragments from genomic DNA of the wild type Col and mutant *cäö* were digested with *Hpa*I (Fermentas, Helsingborg, Sweden) and loaded on a 2.5% agarose gel. The SNP in *cäö* was further validated by standard Sanger sequencing using primers LH1609: CTTGAAGGAAGATAGTGTAACTCGT and LH1324: CCGAATGTATCAGGTCAGCTCTT primers.

### RNA isolation and RT-qPCR

RNA extraction and reverse transcription were performed as described previously [91] with minor modifications. The DNA-free RNA was reverse-transcribed using a RevertAid First Strand cDNA Synthesis Kit (Fermentas, Helsingborg, Sweden) according to manufacturer’s recommendations. Aliquots of the generated cDNA were used as template for PCR with gene-specific primers (S9 Table). Quantitative PCR was performed using gene-specific primers (S9 Table) and SYBR green (Fermentas, Helsingborg, Sweden) on an IQ5 multicolor Real time PCR thermo cycler (BIO-RAD, PA, USA). qPCR reactions were performed in triplicate; gene expression levels were normalized to a *PP2A* control gene, and results were analyzed as described [92].

### Measuring splicing efficiency

Splicing efficiency was measured as described [30] where a primer in an exon was combined with a primer in a neighboring intron (for the unspliced transcript) or covering the splicing junction (for the spliced transcript). For the location of primers for measuring *COOLAIR* splicing see Fig 3A in [30]. RT− controls were always included to confirm absence of genomic DNA contamination.

### Sequence alignment and phylogenetic analysis

Protein sequences of BRR2a homologues were obtained using PSI-BLAST searches, representative organisms from the different eukaryote kingdoms were selected, and their BRR2 amino acid sequences retrieved from protein databases at NCBI. Amino acid sequences were aligned using ClustalW implemented in MEGA5 [93]. Evolutionary analyses were conducted using MEGA5, and a bootstrap Neighbor-Joining Tree was calculated for 1000 bootstrap trials.

### RNA-seq and bioinformatics analysis

For RNA-seq, RNA was isolated from 15-day-old SD-grown Arabidopsis seedlings harvested at 1 h before darkness using the RNeasy Plant Mini Kit (Qiagen). RNA was treated with DNAse I using TURBO DNA-Free Kit (Ambion) and ribosomal RNA was removed using the Ribo-Zero Magnetic kit Plant leaf (cat# MRZPL116, EpiCentre) starting with 1.5μg total RNA. Sequencing libraries were generated from the rRNA depleted RNA using the ScriptSeq v2 RNA seq library prep kit (cat# SSV21124, EpiCentre). Sequencing was performed at an Illumina HiSeq2000 in 100 bp paired-end mode using v3 sequencing chemistry. FastQC v0.10.1 [94] was used to check read quality followed by removal of 10 bp adapter sequences in all samples with trimmomatic v0.32 [95]. Alignments against the Arabidopsis TAIR10 genome were performed with tophat v2.0.10 [96] using default parameters. *De novo* transcriptome assembly of mapped samples was performed using cufflinks v2.1.1. [97], the resulting gtf transcriptome files were merged using cuffmerge v2.1.1. Splicing events were analyzed with ASTALAVISTA v3.0 [49]. RNAseq gene expression counts were generated with HTseq 0.6.1 [98] using the TAIR10 genome annotation. Before the analysis, samples were subjected to batch effect correction using the R package RUVseq v1.0.0 [99] with the empirical control option set to 5,000 genes. Differential gene expression analyses were performed with the R package DESeq2 v1.6.1 [51] using thresholds of p = 0.05 and fold change = 2 for DEG calling after multiple testing correction according to [100]. Intron counts for the first isoform of Arabidopsis transcripts were generated with HTseq 0.6.1. Intron counts were corrected by gene expression using fold change. Differentially retained introns were selected with the DESeq2 package in R using thresholds of p = 0.05 and fold change = 2 after multiple testing correction according to [100]. Further analysis was performed with custom scripts in R v3.1.2. Gene Ontology analysis was performed using GeneCodis [101]. The hypergeometric statistical test with Bonferroni correction was used with a filter requiring three genes as minimum category population. Data are available at GEO (accession number GSE65287).

Data for chromatin properties were taken from the literature: H1.1 and H1.2, [102]; H3K9me2, [103]; H3K4me1, [104]; H3K4me2 and H3K36me2, [105]; H3K4me3 and H3K9ac, [106]; mCG, mCHG and mCHH, [107]; H3K27me3 [108]; H3 and H3K36me3 [109] (H3K9me2, CG, CHG and CHH: seedlings; H3K4me1, H1.1 and H1.2: 3-weeks-old plants; H3K27me3, H3K9ac, H3K4me3, H3K9ac, H3 and H3K36me3: rosette leaves).

It had been reported that budding yeast Brr2p has a particular role in splicing of highly structured introns [57]. Following the method described in [57] and [110], secondary structures were predicted for intron sequences between branch site (BS) and the 3’ss using RNAfold from the Vienna package [111]. Branch sites were predicted as described in [110]. The free energies of the most stable predicted structure for each intron were compared between DRI and unchanged introns using a Wilcoxon signed-rank test. The difference was not significant (p>0.05).

## Supporting Information

S1 Fig Developmental alterations in *cäö*. (A) Rosette diameter of Col and *cäö* plants. Shown are means ± SE (n ≥ 14). (B) Ovule development in Col, homozygous *cäö*^-/-^ and heterozygous *cäö*^+/-^ at 2, 3 and 4 days after emasculation (DAE). Shown are percentage of normally developing ovules (grey) and ovules that lack female gametophyte or are arrested (dark red). Numbers above bars indicate numbers of analyzed ovules.
(PDF)

S2 Fig Genome-wide SNP frequencies in a *cäö* F2 mapping population. (A) SNPs frequencies in sequencing reads were plotted along each chromosome using a bin size of 250 kb. SNPs are caused by reads from the L*er* accession, which was crossed with the *cäö* mutant in Col. A non-recombinant region on the left arm of chromosome 1 with very few L*er* reads is indicated by a brace. Chro., abbreviation for Chromosome. Histograms were generated by the Next-generation mapping tool (Austin et al., 2011 [89]). (B) SNP localization by the Next-Generation Mapping web application. Screenshot of the final stage of region selection and SNP annotation. The sharp delimited peak, at position 7307231 corresponds to the position of a mutation in *AT1G20960*.
(PDF)

S3 Fig Confirmation of the gene mutation by dCAPS and by Sanger sequencing. (A) Gel electrophoretic separation of PCR products (left undigested, right after *Hpa*I digestion). The PCR digestion products were separated by electrophoresis on a 2.5% agarose gel. The mutation in *brr2a*-2 is predicted to inactivate an *Hpa*I recognition site. (B) PCR amplified fragments from Col and *brr2a*-2 genomic DNA were sequenced. Sequences were aligned to Col and *cäö*, and the corresponding amino acids sequences are also listed.
(PDF)

S4 Fig Phylogeny of BRR2 homologues. BRR2 protein sequences of several organisms were aligned and a phylogenetic tree was generated. Branch lengths indicate distances. Numbers on the branch are bootstrap values of confidence in the displayed branches (n = 100). CAA94089.1, AAS78571.1 [*Homo sapiens*] (*Hs*); EAZ28547.1 [*Oryza sativa*] (Os); NP_001116729.1 [*Danio rerio*] (Dr); CAA97301.1, NP_011099.1 [*Saccharomyces_cerevisiae*] (Sc); NP_001185050.1 [*Arabidopsia thaliana*] (BRR2a), NP_181756.1 (BRR2b), NP_200922.2 (BRR2c); NP_648818.3 [*Drosophila melanogaster*] (Dm); NP_796188.2 [*Mus musculus*] (Mm); XP_001757495.1 [*Physcomitrella_patens*] (Pp); XP_002173505.1 [*Schizosaccharomyces japonicus*] (Sc j); XP_002318725.1, XP_002322252.1 [*Populus trichocarpa*] (Pt); XP_002581343.1 [*Schistosoma mansoni*] (Scm); XP_002966396.1, XP_002978166.1, XP_002981317.1 [*Selaginella_moellendorffii*] (Sm); XP_003546783.1, XP_003531516 [*Glycine max*] (Gm); XP_003571468.1 [*Brachypodium distachyon*] (Bd); XP_003595992.1 [*Medicago truncatula*] (Mt); XP_001703610.1 [*Chlamydomonas reinhardtii*] (Cr).
(PDF)

S5 Fig Expression profiles of the Arabidopsis *BRR2* paralogues in different organs. The expression profile of the different *BRR2* paralogues was generated from data in the Arabidopsis eFP Browser (Winter et al., 2007 [38]).
(PDF)

S6 Fig Expression of major *FLC* regulators was not altered in *brr2a*-2. (A) Expression of eight *FLC* repressors. (B) Expression of fifteen *FLC* activators. Quantitative RT-PCR was performed using RNA extracted from 15 day-old seedlings grown under SD conditions at ZT = 7. Relative expression to *PP2a* is shown as mean ± SE (n = 3).
(PDF)

S7 Fig COOLAIR splicing is distorted in *brr2a-2*. (A) Relative splicing of *COOLAIR FLC* antisense RNAs. The three most abundant *COOLAIR* transcripts, class Ii, class Iii and class IIii represent >99% of the total *COOLAIR* (Hornyik et al. 2010 [112]). Abundance of various *COOLAIR* transcripts was analyzed by quantitative RT-PCR using RNA extracted from 15 day-old seedlings grown under LD conditions. Primers were as described by Marquardt et al. 2014 [30]. Abundance relative to *ACTIN2* is shown as mean ± SE (n = 3). (B) Comparison of the splicing efficiency of *COOLAIR* in Col and *brr2a*-2. *COOLAIR* class I was represented by class Ii, class II by class IIi and IIii. Intron retention was computed as (unspliced / (spliced + unspliced)). (C) Comparison of the splicing efficiency of *COOLAIR* in Col and *brr2a*-2 visualized as in (Marquardt et al. 2014 [30]). Splicing ratios (spliced/unspliced) are given normalized to the Col background control.
(PDF)

S8 Fig Expression and intron retention of leaf development genes was altered in *brr2a*-2. (A) Expression of *TEOSINTE BRANCHED 1*, *CYCLOIDEA*, *AND PCF FAMILY 13* (*TCP13*), *KIP-RELATED PROTEIN 6* (*KRP6*), *KRP1*, *TCP24* and *ASYMMETRIC LEAVES 1* (*AS1*) was significantly (p< = 0.05) altered in *brr2a*-2. Expression data are RPKM values from RNA-seq normalized to wild-type levels for each gene. Transcript levels of the remaining 98 leaf development genes (based on GO category GO:0009965) were not significantly altered. (B) Intron 1 of *TCP13* was more retained in *brr2a-2* than in wild type. Shown are RPKM values from RNA-seq normalized to wild-type levels for each intron. Numbers were corrected for changes in transcript amounts to reflect only differential splicing and not transcript abundance. Introns *KRP1*.*I3*, *KRP6*.*I1*, *KRP6*.*I2* and *KRP6*.*I3* did not generate any reads. Values in (A) and (B) are shown as mean ± SE (n = 3).
(PDF)

S9 Fig Confirmation of differentially retained introns. Splicing of 10 genes with increased intron retention in *brr2a*-2 according to the RNA-seq data was analyzed by splicing assays based on quantitative RT-PCR. RNA was extracted from 15 days old Col and *brr2a*-2 seedling grown in LD conditions. Intron retention was analyzed with primers designed to amplify unspliced and spliced transcript, respectively. RNA-seq and qPCR data are compared in a log-log plot. Shown are logarithms of intron retention fold changes (IR FC) between Col and *brr2a*-2. Data points are means of triplicates.
(PDF)

S10 Fig Differentially retained introns in *brr2a*-2 have consensus splice site sequences. The frequency of bases (green, A; blue, C; orange, G; red, T) surrounding the splice sites were calculated and plotted for all (A) and differentially retained introns (B). Sequence logos were created using the SeqLogo R package.
(PDF)

S11 Fig Chromatin properties that do not differ between control introns and introns retained in *brr2a*-2. Box plots show averaged genome-wide bisulfite sequencing and ChIP signals for exons (blue) and introns (green). DRI, differentially retained introns in *brr2a*-2. DRI gene, genes containing at least one differentially retained intron. Thus, the boxes in each plot represent, from left to right, (i) exons from genes that have no DRIs, (ii) exons from genes that have at least one DRI, (iii) normally spliced introns in genes that have at least one DRI, (iv) DRIs, and (v) introns from genes that have no DRIs. Control genes without DRIs were selected to have the same median expression as the DRI genes. No difference is significant (p>0.01) in Wilcoxon signed-rank tests.
(PDF)

S1 Table Amino acid identity (first number) and similarity (second number) between yeast Brr2p and Arabidopsis BRR2 paralogues. (PDF)

S2 Table Sequencing and read mapping details for the RNA-seq experiment. (PDF)

S3 Table Genes with increased transcript levels in *brr2a-2*. (XLSX)

S4 Table Genes with decreased transcript levels in *brr2a-2*. (XLSX)

S5 Table GO categories enriched among genes with decreased transcript levels in *brr2a-2*. (XLSX)

S6 Table GO categories enriched among genes with increased transcript levels in *brr2a-2*. (XLSX)

S7 Table Introns with increased retention in *brr2a-2*. (XLSX)

S8 Table Introns with decreased retention in *brr2a-2*. (XLSX)

S9 Table Lists of primers used. (PDF)
