# Supplementary material for: BRR2a Affects Flowering Time via FLC Splicing
Source: PLoS Genet. 2016 Apr 21;12(4):e1005924. doi: 10.1371/journal.pgen.1005924 (PMC4839602; doi:10.1371/journal.pgen.1005924)
Supplement: S1 Fig — (A) Rosette diameter of Col and cäö plants. Shown are means ± SE (n ≥ 14). (B) Ovule development in Col, homozygous cäö-/- and heterozygous cäö+/- at 2, 3 and 4 days after emasculation (DAE). Shown are percentage of normally developing ovules (grey) and ovules that lack female gametophyte or are arrested (dark red). Numbers above bars indicate numbers of analyzed ovules. (PDF) [file pgen.1005924.s001.pdf]

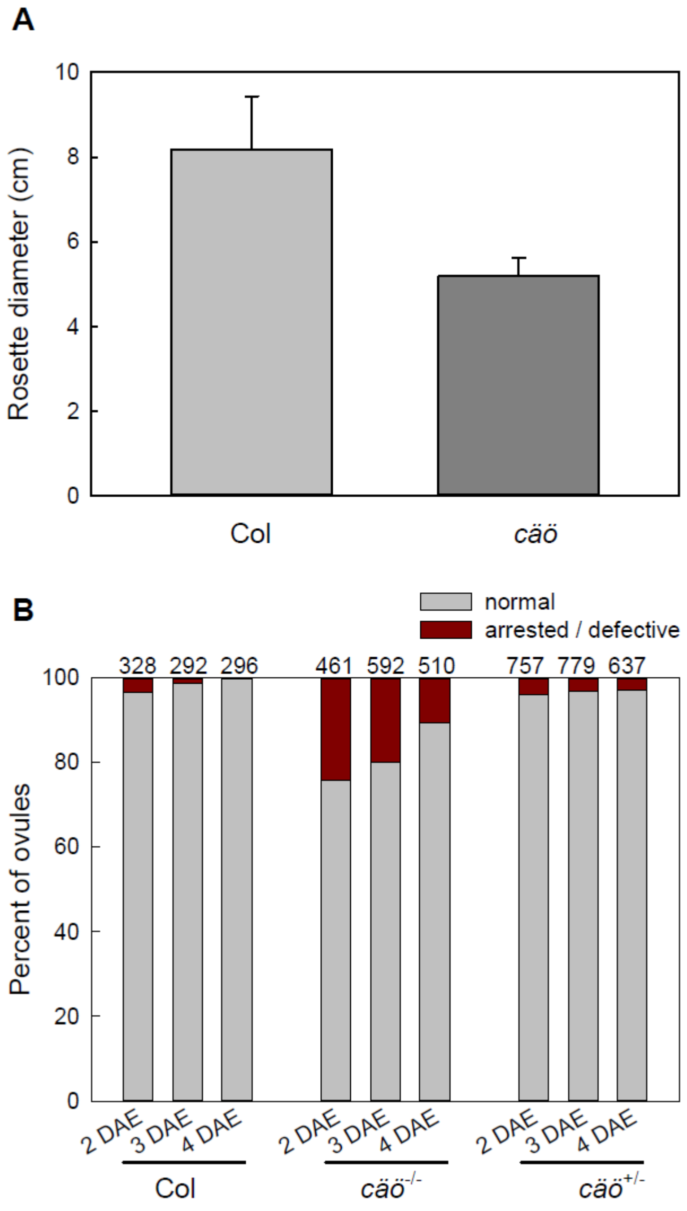

S1 Figure. Developmental alterations in *cäö*. (A) Rosette diameter of Col and *cäö* plants. Shown are means ± SE (n ≥ 14). (B) Ovule development in Col, homozygous *cäö*$^{-/-}$ and heterozygous *cäö*$^{+/-}$ at 2, 3 and 4 days after emasculation (DAE). Shown are percentage of normally developing ovules (grey) and ovules that lack female gametophyte or are arrested (dark red). Numbers above bars indicate numbers of analyzed ovules.
