# Supplementary material for: BRR2a Affects Flowering Time via FLC Splicing
Source: PLoS Genet. 2016 Apr 21;12(4):e1005924. doi: 10.1371/journal.pgen.1005924 (PMC4839602; doi:10.1371/journal.pgen.1005924)
Supplement: S2 Fig — (A) SNPs frequencies in sequencing reads were plotted along each chromosome using a bin size of 250 kb. SNPs are caused by reads from the Ler accession, which was crossed with the cäö mutant in Col. A non-recombinant region on the left arm of chromosome 1 with very few Ler reads is indicated by a brace. Chro., abbreviation for Chromosome. Histograms were generated by the Next-generation mapping tool (Austin et al., 2011 [89]). (B) SNP localization by the Next-Generation Mapping web application. Screenshot of the final stage of region selection and SNP annotation. The sharp delimited peak, at position 7307231 corresponds to the position of a mutation in AT1G20960. (PDF) [file pgen.1005924.s002.pdf]

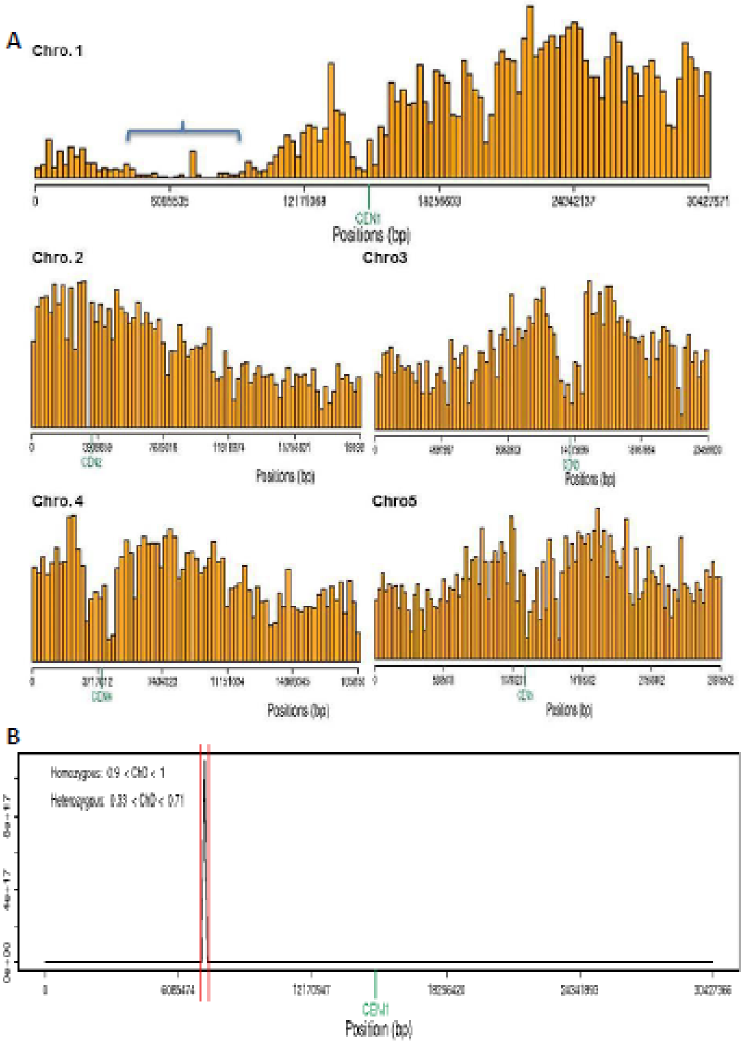

S2 Figure. Genome-wide SNP frequencies in a *cäö* F2 mapping population. (A) SNPs frequencies in sequencing reads were plotted along each chromosome using a bin size of 250 kb. SNPs are caused by reads from the L*er* accession, which was crossed with the *cäö* mutant in Col. A non-recombinant region on the left arm of chromosome 1 with very few L*er* reads is indicated by a brace. Chro., abbreviation for Chromosome. Histograms were generated by the Next-generation mapping tool (Austin et al., 2011). (B) SNP localization by the Next-Generation Mapping web application. Screenshot of the final stage of region selection and SNP annotation. The sharp delimited peak, at position 7307231 corresponds to the position of a mutation in *AT1G20960*.

Austin, R.S., Vidaurre, D., Stamatiou, G., Breit, R., Provart, N.J., Bonetta, D., Zhang, J., Fung, P., Gong, Y., Wang, P.W., McCourt, P. and Guttman, D.S. (2011) Next-generation mapping of Arabidopsis genes. *Plant J* **67**, 715-725.
