# Supplementary material for: BRR2a Affects Flowering Time via FLC Splicing
Source: PLoS Genet. 2016 Apr 21;12(4):e1005924. doi: 10.1371/journal.pgen.1005924 (PMC4839602; doi:10.1371/journal.pgen.1005924)
Supplement: S3 Fig — (A) Gel electrophoretic separation of PCR products (left undigested, right after HpaI digestion). The PCR digestion products were separated by electrophoresis on a 2.5% agarose gel. The mutation in brr2a-2 is predicted to inactivate an HpaI recognition site. (B) PCR amplified fragments from Col and brr2a-2 genomic DNA were sequenced. Sequences were aligned to Col and cäö, and the corresponding amino acids sequences are also listed. (PDF) [file pgen.1005924.s003.pdf]

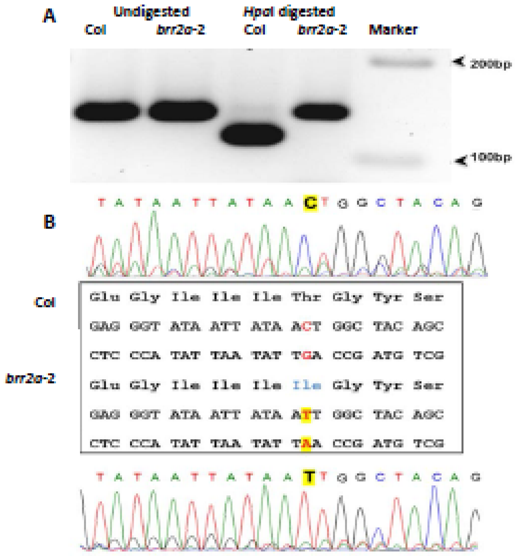

S3 Figure. Confirmation of the gene mutation by dCAPS and by Sanger sequencing. (A) Gel electrophoretic separation of PCR products (left undigested, right after *Hpa*I digestion). The PCR digestion products were separated by electrophoresis on a 2.5% agarose gel. The mutation in *brr2a*-2 is predicted to inactivate an *Hpa*I recognition site. (B) PCR amplified fragments from Col and *brr2a*-2 genomic DNA were sequenced. Sequences were aligned to Col and *cäö*, and the corresponding amino acids sequences are also listed.
