# Supplementary material for: BRR2a Affects Flowering Time via FLC Splicing
Source: PLoS Genet. 2016 Apr 21;12(4):e1005924. doi: 10.1371/journal.pgen.1005924 (PMC4839602; doi:10.1371/journal.pgen.1005924)
Supplement: S4 Fig — BRR2 protein sequences of several organisms were aligned and a phylogenetic tree was generated. Branch lengths indicate distances. Numbers on the branch are bootstrap values of confidence in the displayed branches (n = 100). CAA94089.1, AAS78571.1 [Homo sapiens] (Hs); EAZ28547.1 [Oryza sativa] (Os); NP_001116729.1 [Danio rerio] (Dr); CAA97301.1, NP_011099.1 [Saccharomyces_cerevisiae] (Sc); NP_001185050.1 [Arabidopsia thaliana] (BRR2a), NP_181756.1 (BRR2b), NP_200922.2 (BRR2c); NP_648818.3 [Drosophila melanogaster] (Dm); NP_796188.2 [Mus musculus] (Mm); XP_001757495.1 [Physcomitrella_patens] (Pp); XP_002173505.1 [Schizosaccharomyces japonicus] (Sc j); XP_002318725.1, XP_002322252.1 [Populus trichocarpa] (Pt); XP_002581343.1 [Schistosoma mansoni] (Scm); XP_002966396.1, XP_002978166.1, XP_002981317.1 [Selaginella_moellendorffii] (Sm); XP_003546783.1, XP_003531516 [Glycine max] (Gm); XP_003571468.1 [Brachypodium distachyon] (Bd); XP_003595992.1 [Medicago truncatula] (Mt); XP_001703610.1 [Chlamydomonas reinhardtii] (Cr). (PDF) [file pgen.1005924.s004.pdf]

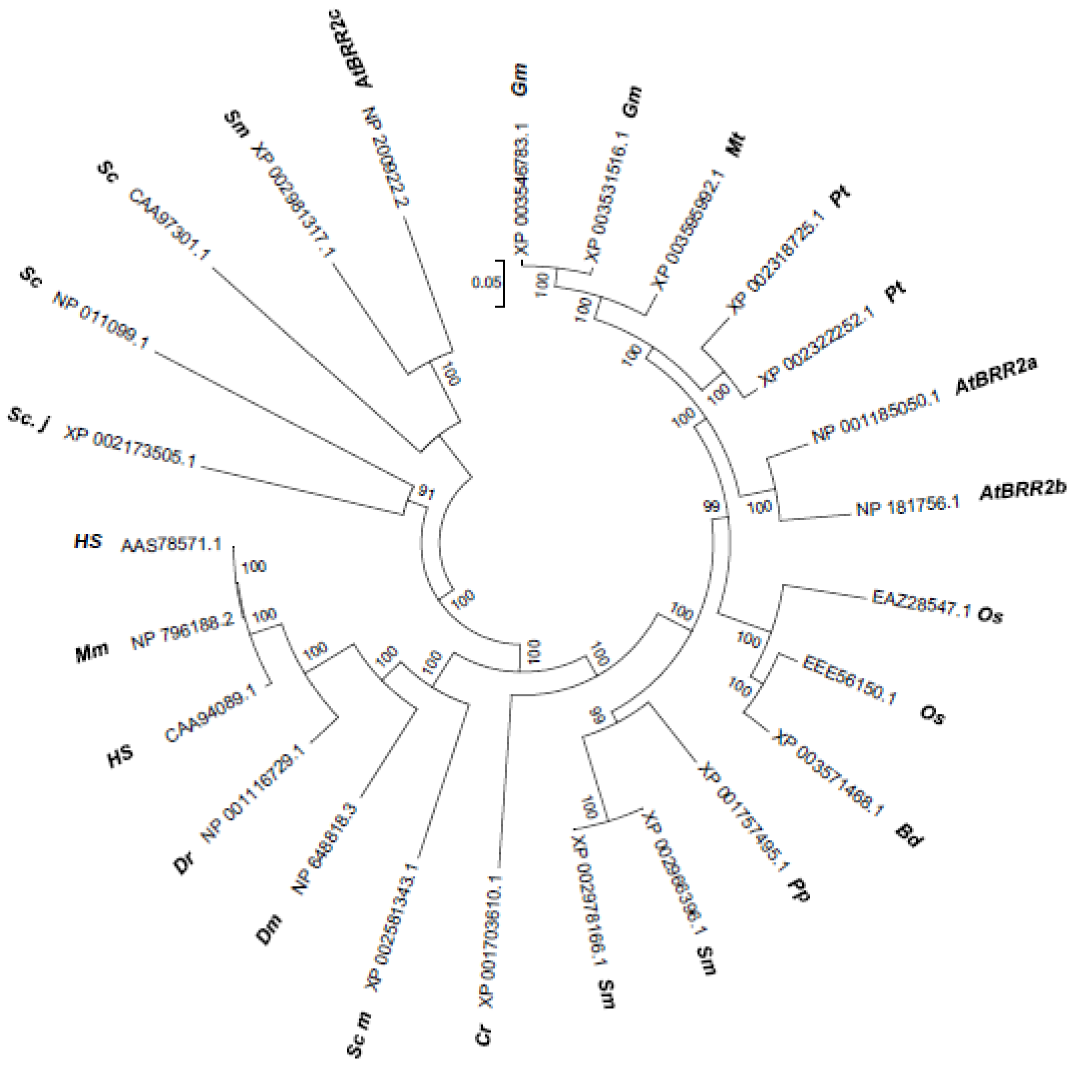

S4 Figure. Phylogeny of BRR2 homologues. BRR2 protein sequences of several organisms were aligned and a phylogenetic tree was generated. Branch lengths indicate distances. Numbers on the branch are bootstrap values of confidence in the displayed branches (n=100). CAA94089.1, AAS78571.1 [*Homo sapiens*] (*Hs*); EAZ28547.1 [*Oryza sativa*] (Os); NP_001116729.1 [*Danio rerio*] (Dr); CAA97301.1, NP_011099.1 [*Saccharomyces_cerevisiae*] (Sc); NP_001185050.1 [*Arabidopsia thaliana*] (BRR2a), NP_181756.1 (BRR2b), NP_200922.2 (BRR2c); NP_648818.3 [*Drosophila melanogaster*] (Dm); NP_796188.2 [*Mus musculus*] (Mm); XP_001757495.1 [*Physcomitrella_patens*] (Pp); XP_002173505.1 [*Schizosaccharomyces japonicus*] (Sc j); XP_002318725.1, XP_002322252.1 [*Populus trichocarpa*] (Pt) ; XP_002581343.1 [*Schistosoma mansoni*] (Scm); XP_002966396.1, XP_002978166.1, XP_002981317.1 [*Selaginella_moellendorffii*] (Sm) ; XP_003546783.1, XP_003531516 [*Glycine max*] (Gm); XP_003571468.1 [*Brachypodium distachyon*] (Bd); XP_003595992.1 [*Medicago truncatula*] (Mt); XP_001703610.1 [*Chlamydomonas reinhardtii*] (Cr).
