# Supplementary material for: BRR2a Affects Flowering Time via FLC Splicing
Source: PLoS Genet. 2016 Apr 21;12(4):e1005924. doi: 10.1371/journal.pgen.1005924 (PMC4839602; doi:10.1371/journal.pgen.1005924)
Supplement: S5 Fig — The expression profile of the different BRR2 paralogues was generated from data in the Arabidopsis eFP Browser (Winter et al., 2007 [38]). (PDF) [file pgen.1005924.s005.pdf]

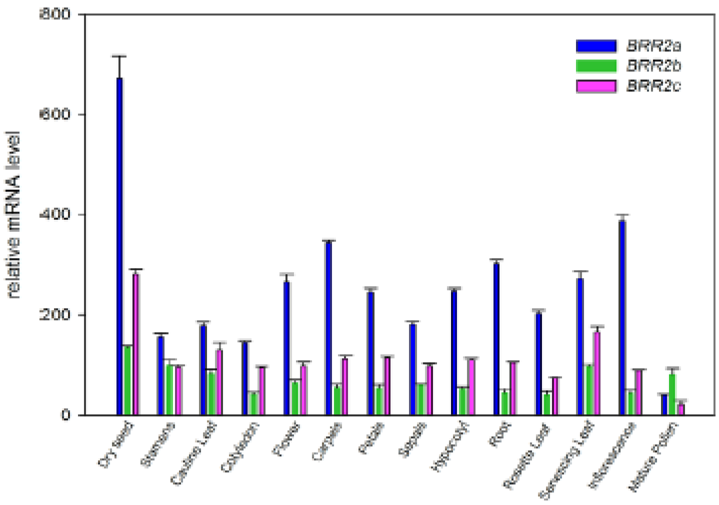

S5 Figure. Expression profiles of the Arabidopsis *BRR2* paralogues in different organs. The expression profile of the different *BRR2* paralogues was generated from data in the Arabidopsis eFP Browser (Winter et al., 2007).

Winter, D., Vinegar, B., Nahal, H., Ammar, R., Wilson, G.V. and Provart, N.J. (2007) An "Electronic Fluorescent Pictograph" browser for exploring and analyzing large-scale biological data sets. *PLoS One* **2**.
