# Supplementary material for: BRR2a Affects Flowering Time via FLC Splicing
Source: PLoS Genet. 2016 Apr 21;12(4):e1005924. doi: 10.1371/journal.pgen.1005924 (PMC4839602; doi:10.1371/journal.pgen.1005924)
Supplement: S6 Fig — (A) Expression of eight FLC repressors. (B) Expression of fifteen FLC activators. Quantitative RT-PCR was performed using RNA extracted from 15 day-old seedlings grown under SD conditions at ZT = 7. Relative expression to PP2a is shown as mean ± SE (n = 3). (PDF) [file pgen.1005924.s006.pdf]

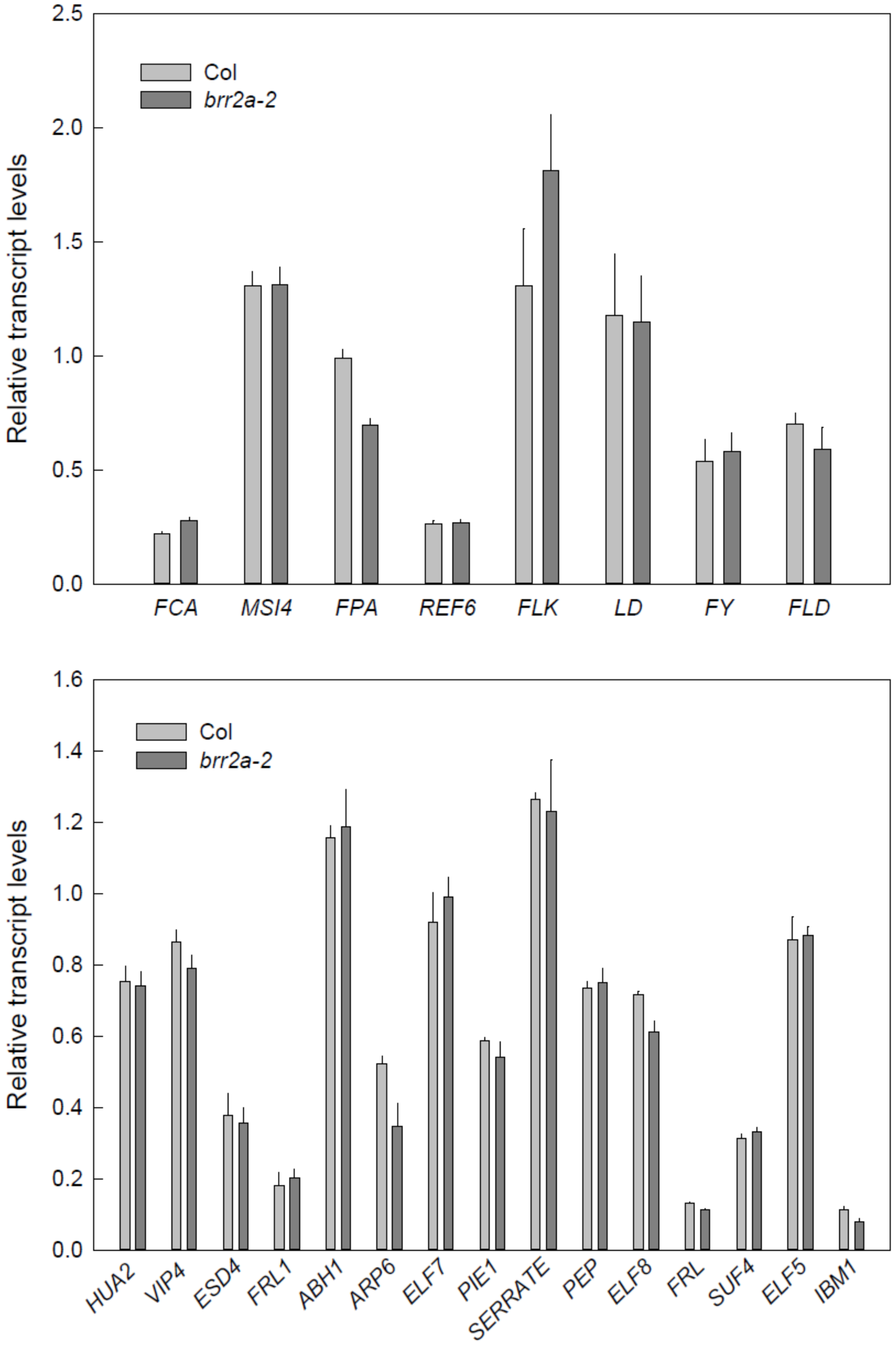

S6 Figure. Expression of major *FLC* regulators was not altered in *brr2a*-2. (A) Expression of eight *FLC* repressors. (B) Expression of fifteen *FLC* activators. Quantitative RT-PCR was performed using RNA extracted from 15 day-old seedlings grown under SD conditions at ZT = 7. Relative expression to *PP2a* is shown as mean ± SE (n = 3).
