# Supplementary material for: BRR2a Affects Flowering Time via FLC Splicing
Source: PLoS Genet. 2016 Apr 21;12(4):e1005924. doi: 10.1371/journal.pgen.1005924 (PMC4839602; doi:10.1371/journal.pgen.1005924)
Supplement: S7 Fig — (A) Relative splicing of COOLAIR FLC antisense RNAs. The three most abundant COOLAIR transcripts, class Ii, class Iii and class IIii represent >99% of the total COOLAIR (Hornyik et al. 2010 [112]). Abundance of various COOLAIR transcripts was analyzed by quantitative RT-PCR using RNA extracted from 15 day-old seedlings grown under LD conditions. Primers were as described by Marquardt et al. 2014 [30]. Abundance relative to ACTIN2 is shown as mean ± SE (n = 3). (B) Comparison of the splicing efficiency of COOLAIR in Col and brr2a-2. COOLAIR class I was represented by class Ii, class II by class IIi and IIii. Intron retention was computed as (unspliced / (spliced + unspliced)). (C) Comparison of the splicing efficiency of COOLAIR in Col and brr2a-2 visualized as in (Marquardt et al. 2014 [30]). Splicing ratios (spliced/unspliced) are given normalized to the Col background control. (PDF) [file pgen.1005924.s007.pdf]

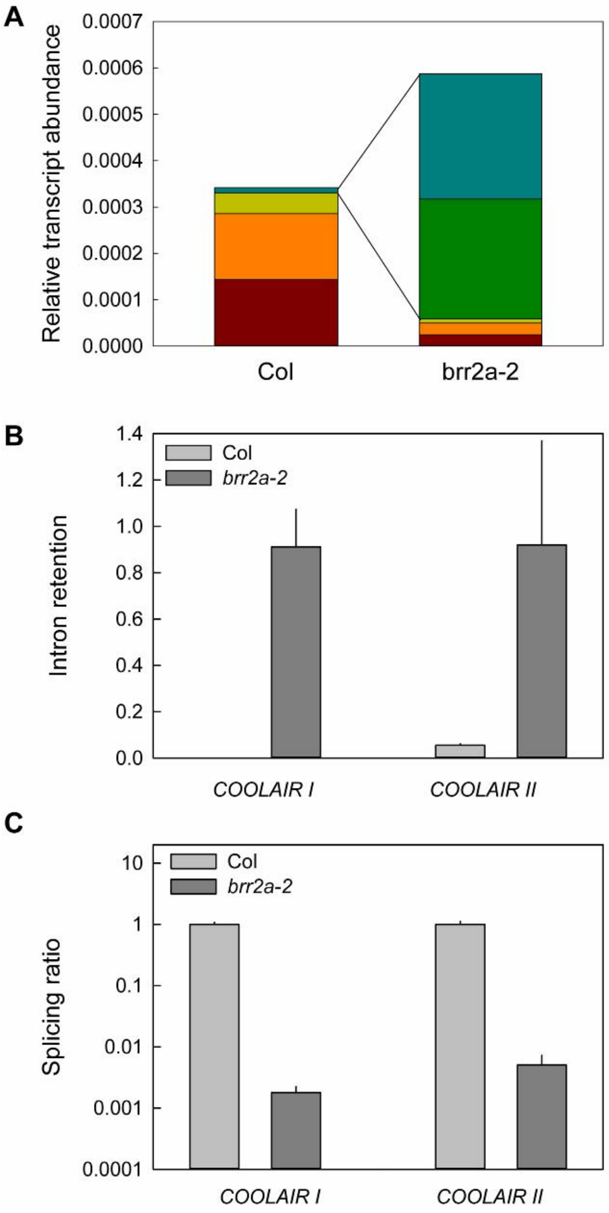

S7 Figure. COOLAIR splicing is distorted in *brr2a-2*. (A) Relative splicing of *COOLAIR FLC* antisense RNAs. The three most abundant *COOLAIR* transcripts, class Ii, class Iii and class IIii represent >99% of the total *COOLAIR* (Hornyik et al. 2010). Abundance of various *COOLAIR* transcripts was analyzed by quantitative RT-PCR using RNA extracted from 15 day-old seedlings grown under LD conditions. Primers were as described by Marquardt et al. 2014. Abundance relative to *ACTIN2* is shown as mean ± SE (n = 3). (B) Comparison of the splicing efficiency of *COOLAIR* in Col and *brr2a*-2. *COOLAIR* class I was represented by class Ii, class II by class IIi and IIii. Intron retention was computed as (unspliced / (spliced + unspliced)). (C) Comparison of the splicing efficiency of *COOLAIR* in Col and *brr2a*-2 visualized as in (Marquardt et al. 2014). Splicing ratios (spliced/unspliced) are given normalized to the Col background control.

Hornyik, C., Terzi, L.C. and Simpson, G.G. (2010) The spen family protein FPA controls alternative cleavage and polyadenylation of RNA. *Dev Cell* **18:** 203-213.

Marquardt, S., Raitskin, O., Wu, Z., Liu, F., Sun, Q. and Dean, C. (2014) Functional consequences of splicing of the antisense transcript COOLAIR on FLC transcription. *Mol Cell* **54:** 156-165.
