# Supplementary material for: BRR2a Affects Flowering Time via FLC Splicing
Source: PLoS Genet. 2016 Apr 21;12(4):e1005924. doi: 10.1371/journal.pgen.1005924 (PMC4839602; doi:10.1371/journal.pgen.1005924)
Supplement: S8 Fig — (A) Expression of TEOSINTE BRANCHED 1, CYCLOIDEA, AND PCF FAMILY 13 (TCP13), KIP-RELATED PROTEIN 6 (KRP6), KRP1, TCP24 and ASYMMETRIC LEAVES 1 (AS1) was significantly (p< = 0.05) altered in brr2a-2. Expression data are RPKM values from RNA-seq normalized to wild-type levels for each gene. Transcript levels of the remaining 98 leaf development genes (based on GO category GO:0009965) were not significantly altered. (B) Intron 1 of TCP13 was more retained in brr2a-2 than in wild type. Shown are RPKM values from RNA-seq normalized to wild-type levels for each intron. Numbers were corrected for changes in transcript amounts to reflect only differential splicing and not transcript abundance. Introns KRP1.I3, KRP6.I1, KRP6.I2 and KRP6.I3 did not generate any reads. Values in (A) and (B) are shown as mean ± SE (n = 3). (PDF) [file pgen.1005924.s008.pdf]

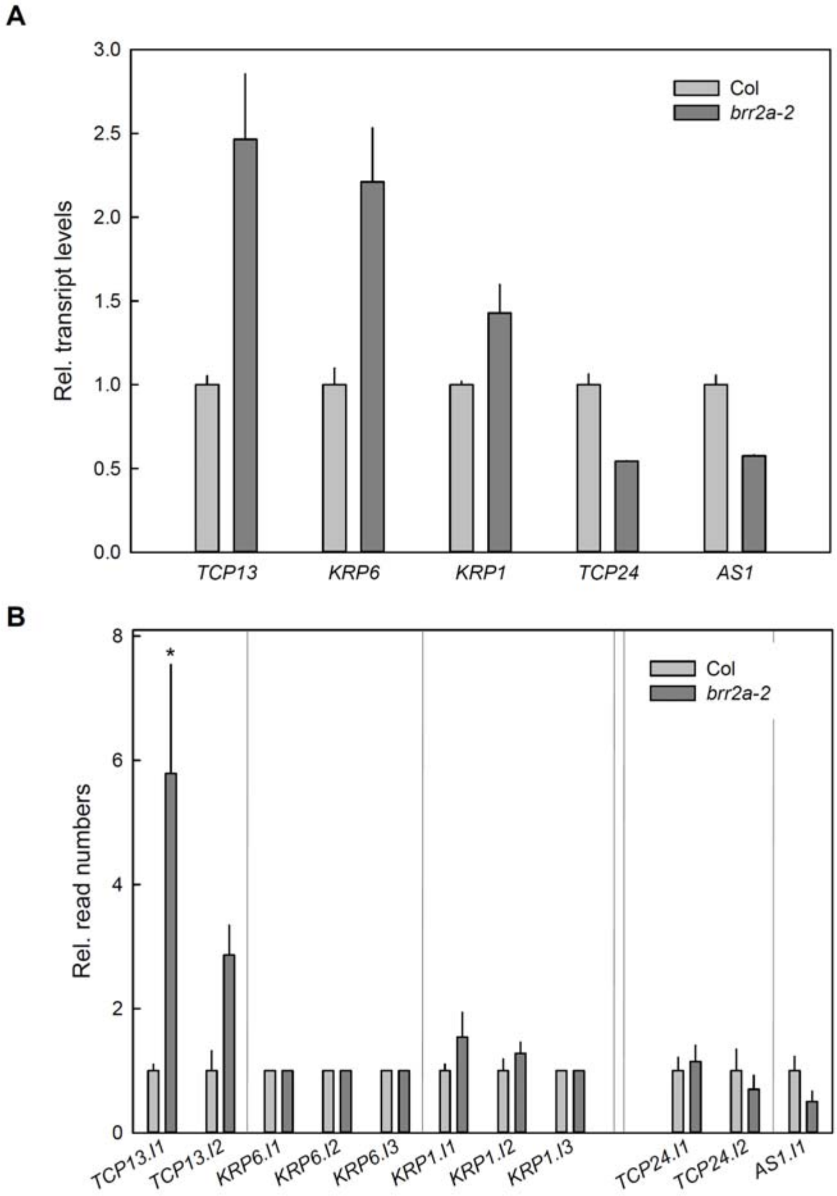

S8 Figure. Expression and intron retention of leaf development genes was altered in *brr2a*-2. (A) Expression of *TEOSINTE BRANCHED 1, CYCLOIDEA, AND PCF FAMILY 13* (*TCP13*), *KIP-RELATED PROTEIN 6* (*KRP6*), *KRP1*, *TCP24* and *ASYMMETRIC LEAVES 1* (*AS1*) was significantly (p<=0.05) altered in *brr2a*-2. Expression data are RPKM values from RNA-seq normalized to wild-type levels for each gene. Transcript levels of the remaining 98 leaf development genes (based on GO category GO:0009965) were not significantly altered. (B) Intron 1 of *TCP13* was more retained in *brr2a-2* than in wild type. Shown are RPKM values from RNA-seq normalized to wild-type levels for each intron. Numbers were corrected for changes in transcript amounts to reflect only differential splicing and not transcript abundance. Introns *KRP1.I3*, *KRP6.I1*, *KRP6.I2* and *KRP6.I3* did not generate any reads. Values in (A) and (B) are shown as mean ± SE (n = 3).
