# Supplementary material for: BRR2a Affects Flowering Time via FLC Splicing
Source: PLoS Genet. 2016 Apr 21;12(4):e1005924. doi: 10.1371/journal.pgen.1005924 (PMC4839602; doi:10.1371/journal.pgen.1005924)
Supplement: S9 Fig — Splicing of 10 genes with increased intron retention in brr2a-2 according to the RNA-seq data was analyzed by splicing assays based on quantitative RT-PCR. RNA was extracted from 15 days old Col and brr2a-2 seedling grown in LD conditions. Intron retention was analyzed with primers designed to amplify unspliced and spliced transcript, respectively. RNA-seq and qPCR data are compared in a log-log plot. Shown are logarithms of intron retention fold changes (IR FC) between Col and brr2a-2. Data points are means of triplicates. (PDF) [file pgen.1005924.s009.pdf]

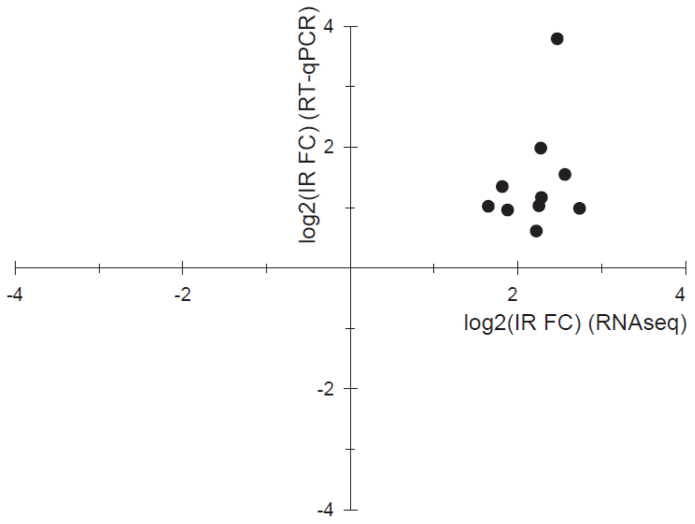

S9 Figure. Confirmation of differentially retained introns. Splicing of 10 genes with increased intron retention in *brr2a*-2 according to the RNA-seq data was analyzed by splicing assays based on quantitative RT-PCR. RNA was extracted from 15 days old Col and *brr2a*-2 seedling grown in LD conditions. Intron retention was analyzed with primers designed to amplify unspliced and spliced transcript, respectively. RNA-seq and qPCR data are compared in a log-log plot. Shown are logarithms of intron retention fold changes (IR FC) between Col and *brr2a*-2. Data points are means of triplicates.
