# Supplementary material for: BRR2a Affects Flowering Time via FLC Splicing
Source: PLoS Genet. 2016 Apr 21;12(4):e1005924. doi: 10.1371/journal.pgen.1005924 (PMC4839602; doi:10.1371/journal.pgen.1005924)
Supplement: S10 Fig — The frequency of bases (green, A; blue, C; orange, G; red, T) surrounding the splice sites were calculated and plotted for all (A) and differentially retained introns (B). Sequence logos were created using the SeqLogo R package. (PDF) [file pgen.1005924.s010.pdf]

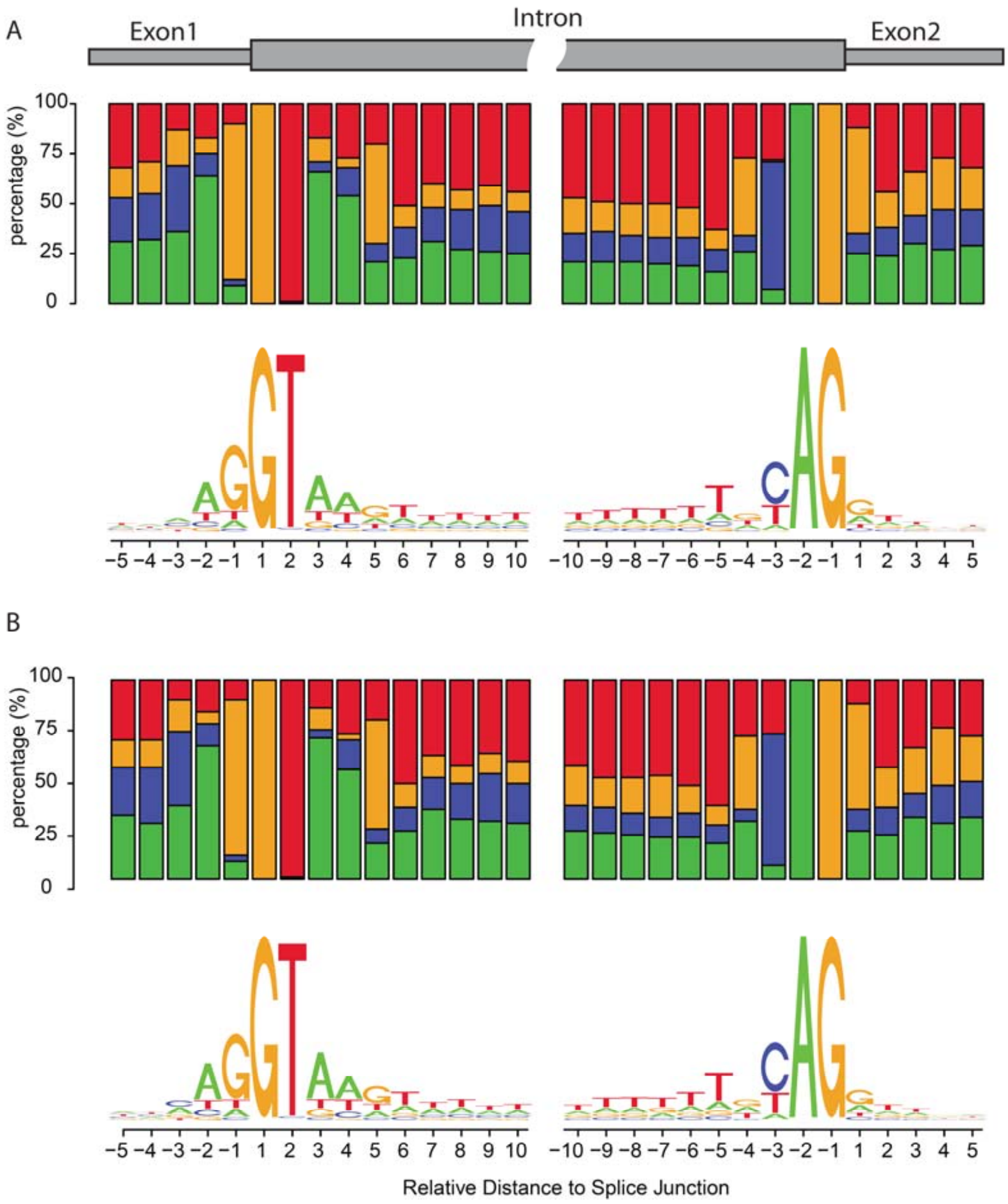

S10 Figure. Differentially retained introns in *brr2a*-2 have consensus splice site sequences. The frequency of bases (green, A; blue, C; orange, G; red, T) surrounding the splice sites were calculated and plotted for all (A) and differentially retained introns (B). Sequence logos were created using the SeqLogo R package.
