# Supplementary material for: BRR2a Affects Flowering Time via FLC Splicing
Source: PLoS Genet. 2016 Apr 21;12(4):e1005924. doi: 10.1371/journal.pgen.1005924 (PMC4839602; doi:10.1371/journal.pgen.1005924)
Supplement: S11 Fig — Box plots show averaged genome-wide bisulfite sequencing and ChIP signals for exons (blue) and introns (green). DRI, differentially retained introns in brr2a-2. DRI gene, genes containing at least one differentially retained intron. Thus, the boxes in each plot represent, from left to right, (i) exons from genes that have no DRIs, (ii) exons from genes that have at least one DRI, (iii) normally spliced introns in genes that have at least one DRI, (iv) DRIs, and (v) introns from genes that have no DRIs. Control genes without DRIs were selected to have the same median expression as the DRI genes. No difference is significant (p>0.01) in Wilcoxon signed-rank tests. (PDF) [file pgen.1005924.s011.pdf]

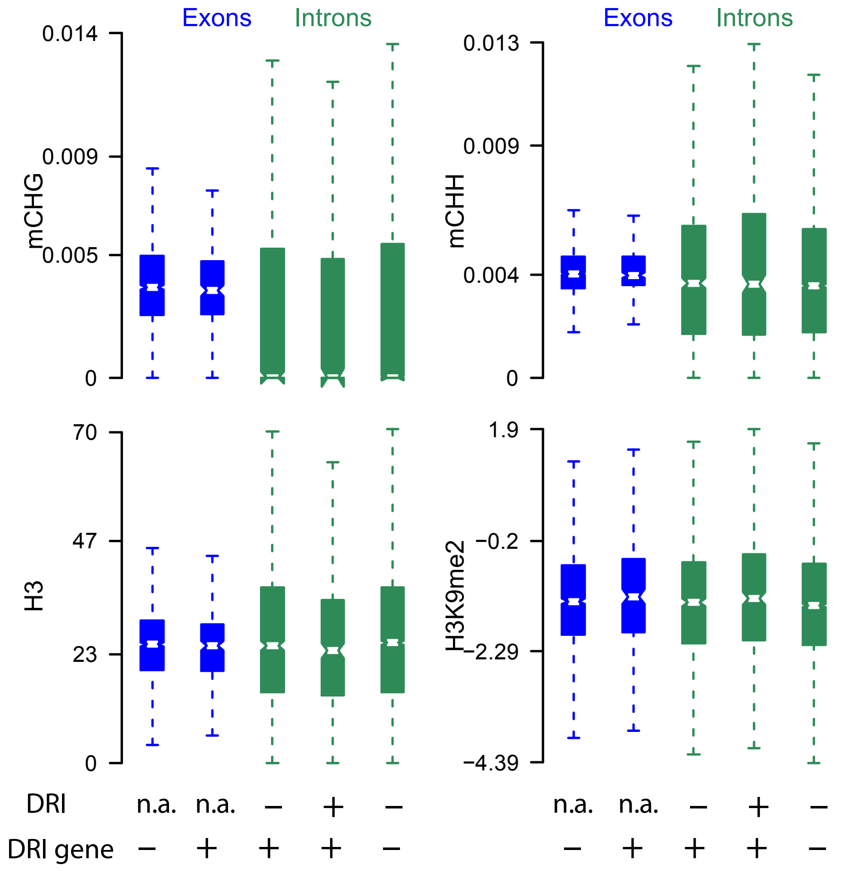

S11 Figure. Chromatin properties that do not differ between control introns and introns retained in *brr2a*-2. Box plots show averaged genome-wide bisulfite sequencing and ChIP signals for exons (blue) and introns (green). DRI, differentially retained introns in *brr2a*-2. DRI gene, genes containing at least one differentially retained intron. Thus, the boxes in each plot represent, from left to right, (i) exons from genes that have no DRIs, (ii) exons from genes that have at least one DRI, (iii) normally spliced introns in genes that have at least one DRI, (iv) DRIs, and (v) introns from genes that have no DRIs. Control genes without DRIs were selected to have the same median expression as the DRI genes. No difference is significant (p>0.01) in Wilcoxon signed-rank tests.
