# Supplementary material for: BRR2a Affects Flowering Time via FLC Splicing
Source: PLoS Genet. 2016 Apr 21;12(4):e1005924. doi: 10.1371/journal.pgen.1005924 (PMC4839602; doi:10.1371/journal.pgen.1005924)
Supplement: S1 Table — (PDF) [file pgen.1005924.s012.pdf]

S1 Table. Amino acid identity (first number) and similarity (second number) between yeast Brr2p and Arabidopsis BRR2 paralogues

	AT2G42270 BRR2b	AT5G61140 BRR2c	Brr2p
AT1G20960 BRR2a	82 / 91	40 / 59	38 / 60
AT2G42270 BRR2b		39 / 58	38 /59
AT5G61140 BRR2c			30 /52
