# Supplementary material for: BRR2a Affects Flowering Time via FLC Splicing
Source: PLoS Genet. 2016 Apr 21;12(4):e1005924. doi: 10.1371/journal.pgen.1005924 (PMC4839602; doi:10.1371/journal.pgen.1005924)
Supplement: S2 Table — (PDF) [file pgen.1005924.s013.pdf]

S2 Table. Sequencing and read mapping details for the RNA-seq experiment.

	Col 1	Col 2	Col 3	*brr2a-2 1*	*brr2a-2 2*	*brr2a-2 3*
Raw reads	39,540,006	27,254,569	23,504,681	28,675,398	26,246,034	22,744,722
Mapped reads	32,769,149	23,956,774	21,621,461	25,881,577	22,035,030	20,963,981
	83%	*88%*	*92%*	*90%*	*84%*	*92%*
Reads counted in genes	29,830,638	21,739,180	19,468,030	23,184,873	19,969,590	18,692,830
	91%	*91%*	*90%*	*90%*	*91%*	*89%*
Reads counted in introns	1,065,088	600,319	561,146	1,044,438	848,684	739,835
	3.3%	*2.5%*	*2.6%*	*4.0%*	*3.9%*	*3.5%*
