# Supplementary material for: BRR2a Affects Flowering Time via FLC Splicing
Source: PLoS Genet. 2016 Apr 21;12(4):e1005924. doi: 10.1371/journal.pgen.1005924 (PMC4839602; doi:10.1371/journal.pgen.1005924)
Supplement: S9 Table — (PDF) [file pgen.1005924.s020.pdf]

S9 Table. Lists of primers used.

**Gene-specific primers used for splicing assays**	
**Gene / Primer position**	**Primer ID/Primer sequence**
FLC_I[*]5_R[*]	LH2039: AATATGTGTGCAAGCTCGTTAAG
FLC_E[*]5_F[*]	LH2040: AACTCATGTTGAAGCTTGTTGAG
FLC_E6_R	LH2041: CCTGGTTCTCTTCTTTCAGCAT
FLC_E1_F	LH1801: TTCTCCAAACGTCGCAACGGTCTC
FLC_I1-R	LH1802: CTCAGAAAAGTAAAAGAGCACAAAACAG
FLC_E2-R	LH1803: CATGCTGTTTCCCATATCGATCAAG
FLC_I6-2-F	LH1804: CCGAGTGGCTCAGTTCCAACTCCA
FLC_E4-1-F	LH1805: ACACCTTGAGACTGCCCTCTCCGT
FLC_E7-1-R	LH1806: ACGCTCGCCCTTATCAGCGGAA
MAF1_E1_F	LH2042: CGCTGTTGTCGTCGTATCT
MAF1_I1_R	LH2043: AAATCGAGTGAGACCCAGAAA
MAF1_E2_R	LH2044: TGAGGTTTCTCCGGCTTGA
AG_E1_F	LH2957: CGTGGTCGTCTCTATGAGTAC
AG_E2_R	LH2958: GCCTTCTTGTACCTCTCAATAGT
AG_I1_F	LH2959: GGTAGTTAATAGGTCTAATTAACTTAT
AG_I1_R	LH2960: GTGCAAGTGGTCGATTTGA
SEP3_E1_F	LH2961: GAGGAAAGCTGTACGAGTTTTGCAG
SEP3_E2_R	LH2962: CCTCTCCAGTGTCCGAAGC
SEP3_I2-2_F	LH2964: GATGAGTAAGATTAATGGAAGAAATGATG
COOLAIR_I1_FT[*]	LH2965: CTGCTGGACAAATCTCCGACAATC
COOLAIR_I1_spliced_F	LH2966: GACAAATCTCCGACAATCTTCC
COOLAIR_I1_spliced_R	LH2967: CTCACACGAATAAGGTGGCTAAT
COOLAIR_I1_unspliced_F	LH2968: CGACAATCTTCCGGTGACTCT
COOLAIR_I1_unspliced_F	LH2969: TACAAACGCTCGCCCTTATC
COOLAIR_classII-1/2_F	LH2970: CTCCTCCGGCGATAAGTA
COOLAIR_classII-1_R	LH2971: CTCACACGAATAAGAAAAGTAAAA
COOLAIR_classII-2_R	LH2972: ACGATAATCATAGAAAAGTAAAAGAGC
COOLAIR_classII_unspliced_F	LH2973: TCGCTCTTCTCGTCGTCTC
COOLAIR_classII unspliced_R	LH2974: AAAACACAAACAAACACAGAACC
AT5G53740_I6_F	LH3139:GGTCATCACTCCTCAACATTCA
AT5G53740_I6_R	LH3140: GGAAACATTTTTGGCCAACAC
AT5G53740_E6_F	LH3141: TAAGTGATGTTTCCGTTGAATCCAT
AT5G53740_E7_R	LH3142: AGGTTTCGTGTGTGTTGGTG
AT1G23310_I3_F	LH3143: CTCGCCAGGTATGTGTGTATAG
AT1G23310_I3_R	LH3144: AGCGCAACCACCTGTAATTG
AT1G23310_E3_F	LH3145: CCAGGTGGTTGCGCTTTG
AT1G23310_E4_R	LH3146: CCGCCTGAAGTCAAGGAAAG
AT1G67090_I2_F	LH3147: GAATTCGAGTTGGAGGTAATTAAACA
AT1G67090_I2_R	LH3148: ACCAAATTTTCCTAAGATCTAGCTAGT
AT1G67090_E2_F	LH3149: GAGTTGGAGACGGATTTGTGTACCG
AT1G67090_E3_R	LH3150: GTCCAGTACCGTCCATCATAGT

AT3G57550_I9_F	LH3151: CCTGTGAATGGTGTAGAAGGTAATA
AT3G57550_E10_R	LH3152: TGTTCTTGGTTCCTTCTCCTGT
AT3G57550_E9_F	LH3153: GGTGTAGAAGCAGTAGAAGGGAT
AT1G59990_I1_F	LH3154: CGATAGACCATCTCTTACTCAGGTA
AT1G59990_I1_R	LH3155: TCCCAATCAACAAATGCTTCTCT
AT1G59990_E1_F	LH3156: TAGACCATCTCTTACTCAGGCTGT
AT1G59990_E2_R	LH3157: TCTGCTGCAACAATCACATCT
AT5G02120_I2_F	LH3158: GTACTTTCATCGTCGAACTGGTGAG
AT5G02120_I2_R	LH3159: TCAGTTTGGTTTCGTTGTATGTT
AT5G02120_E2_F	LH3160: GTACTTTCATCGTCGAACTGATTC
AT5G02120_E3_R	LH3223:GGAAGATCGAGTCCTTTCCCA
AT3G56990_I14_F	LH3224:GGTTCGATGTTCCAAAATCCGGT
AT3G56990_I14_R	LH3225: GTTTGATACGATGTGAAATAGAGCA
AT3G56990_E14_F	LH3226: TCCGGACTTCCAAATTGATAAGGACT
AT3G56990_E15_R	LH3227: TCGAAGAAGCAACAGGGTGT
AT3G56990_I11_F	LH3228: CCATTCCAGTGATGTCACCAT
AT3G56990_I11_R	LH3229: TGTCTGTGCACTTTCCTCCA
AT3G56990_E11_F	LH3230: CTCCTCTTGAAAATCTAACGGAAGAG
AT3G56990_E12_R	LH3231: CCAGATCTTCCATTGCCAAA
AT1G42970_I3_F	LH3232: GTGGTGGTGTCAAGAATGTAAGTATG
AT1G42970_I3_R	LH3233: GGATGCCTGCAAATTTCATC
AT1G42970_E3_F	LH3234: GTGTCAAGAATGCATCCCACT
AT1G42970_E4_R	LH3235: GGTTCCAAGCATGGAGTCAT
AT3G47590_I4_F	LH3236: AAAGAGTGCAGGTGAGACTCG
AT3G47590_I4_R	LH3237: ACCGTCAAGACCCTGTCATT
AT3G47590_E4_F	LH3238: AGGGTCTTGACGGTTCATGG
AT3G47590_E5_R	LH3239: GCAAACTCCTTCGCATCTTC
AT3G57550_I8_F	LH3240: CCTTGAGGAATGCTACAAGACC
AT3G57550_I8_R	LH3241: CCATTGTTGCTTGAAATGGA
AT3G57550_E8_F	LH3242: CCTTAAGAATCTCTTGGGGATTAAC
AT3G57550_E9_R	LH3243: TCTACACCATTCACAGGAGCA
AT3G57570_I2_F	LH3244: CGAAGCCATGAGTGAAATTCT
AT3G57570_I2_R	LH3245: GACCGGACATTACAACAATCAA
AT3G57570_E2_F	LH3246: GTACCCATTATCGAACATACATTAAG
AT3G57570_E3_R	LH3247: GAACTTCATCTTGAAATAGCCAAT
AT3G57570_I4_F	LH3248: TGGGTATAAATATCATCTCCTGCT
AT3G57570_I4_R	LH3249: CAACAGCTCAGAACAAAGTGG
AT3G57570_E4_F	LH3250: TTTGTCAATGGATGGATATGAGGT
AT3G57570_E5_R	LH3251: CCTCTTTGCTAAGGCTCCAG
AT5G64040_I2_F	LH3252: GATCTTGCCAAGCAAAAGGT
AT5G64040_I2_R	LH3253: GAAAACGAACAAGAACATCACAA
AT5G64040_E2_F	LH3254: CCAAGCAAAAGTTCCATTTATCTC
AT5G64040_E3_R	LH3255: TCCAGAAAACATTGGAACCAC

*I: Intron, E: Exon, F: Forward primer, R: Reverse primer, FT: First strand primer

Gene-specific primers used for RT-qPCR	
**Gene ID, Alias**	**Primer ID/Primer sequence**
AT2G10930, PP2A	DK053: TCTTAATTTGAATCGGTTACTAGG<br>DK054: CATACATAACAATTTCACAAGCA
AT1G20960, BRR2a	LH1556: GCCCTTGCAAAAGACGTAGT<br>LH1557: AGCAGCCGAATGTATCAGGT
AT1G65480, FT	LH0292: ACAACTGGAACAACCTTTGGCAATG<br>LH0293: ACTGTTTGCCTGCCAAGCTGTC
AT2G45660, SOC1	SUPERSOC1-F452: AGCAGCTCAAGCAAAAGGAG<br>SUPERSOC1-R541: TTGACCAAACTTCGCTTTCA
AT5G10140, FLC	LH0304: GACTGCCCTCTCCGTGACTA<br>LH0305: TTCTCAACAAGCTTCAACATGAG
AT1G77080, MAF1	SUPERMAF3-SALKLP: TTCGGAATTATCTTCCACACAA<br>LH0780: CGGACAGAGCAGTCTCAAGTT
AT5G65050, MAF2	LH0781: GGCTCCGGAAAACTCTACAA<br>LH0535: GAGTGGCAGATAATTCCGAG
AT5G65060, MAF3	LH0755: GACGTTTCTGGTTATAGAAGGTG<br>LH0827: TTACTTGAGCAGCGAAAGAGTC
AT5G65070, MAF4	LH0541: GGAGCTTGTGAAGACCCATC<br>LH0542: TTCCCCATCTTTATAAGCTGG
AT5G65080, MAF5	LH0543: TCCACCGGCAAACTCTACA<br>LH0544: AAACGACTGATGATCTTGGC
AT4G16280, FCA δ γ	LH1747: GAACTGGACAGCAGCAAGGCTGTTG<br>LH1748: AGGGTGCCTATGCGTTCTCTCTCC
AT2G19520, MSI4	MSI4-838F: ACGAAGGTTGAAAAAGCGCA<br>MSI4-950R: CGATCAAACAACCGGACAGTG
AT2G43410, FPA	LH1700: TGGGTGTCGATGAGAGGTCAT<br>LH1701: CAATTGACGACCTCAGGCAGT
AT3G10390, FLD	LH1974: CTCTCTTGATCGCGTTGGTT<br>LH1975: GGACATTTATTCCTTGAGGTTCA
AT4G02560, LD	LH1702: CAACTAATCCTGGAATGAGTGG<br>LH1703: GGTTGTTGAGATTGGTTGTTGT
AT5G13480, FY	LH1704: GATGCCTGGATCAATGGGAATG<br>LH1705: TGCTGCTGTTGGAAAGGGTTGT
AT3G04610, FLK	LH1706: CCACCAATGGTCGCTCAGCAAG<br>LH1707: ATCCGTAGCGTATCCTCCAGGCG
AT3G48430, REF6	LH1708: CGGAATACCGTGTTGCAGGTTAG<br>LH1709: CCGGATAGCAGCATCTTTAGCCA
AT4G15880, ESD4	LH1710: GCTCTGGCAAAATACATGGGTGATGAAG<br>LH1711: GAAGTATGGCATGTGTTCCTGGCTGAA
AT5G16320, FRL1	LH1712: TCACAAGTTCCAGTTCCGAGTCAGCA<br>LH1713: TTGAGGCGGCAACACATATCCAGTTT
AT5G16320, HUA2	LH1714: CTTACCCTTTCCCACCTCAACGTGAT<br>LH1715: ATCTGGGGAGCAGTATGACCTGGAAT
AT5G61150, VIP4	LH1716: GCCAGACCGTCAAGACGTCAAATGGAG<br>LH1717: ATCCTTCTCTGCCCTACCTCCCGCAA

AT3G07610, IBM1	LH2045: CCCGAGAATCATTGGTTACAG<br>LH2046: CACCGTTCAACAATCCTATCG
AT2G13540, ABH1	LH1777: CGGCTCCCAGATCCAACTAAAGTG<br>LH1778: CTCCATGTGAGGCCATATCTCGCT
AT3G33520, ARP6	LH1779: GTACCTGAGACGTTATTCCAGCCTGC<br>LH1780: CTCCATACACCTAGTATGGGGTCCTC
AT5G62640, ELF5	LH1783: CACACCTGAGCTTACATCCATGGTCCC<br>LH1784: GTGCGCCAAGAGCTTTCATGTCCTCTA
AT1G79730, ELF7	LH1785: CCTGGCACATACCTGGTATCATTTGAC<br>LH1786: ACCAACCCTAGAAGAGTAAACCCCTGA
AT2G06210, ELF8	LH1787: GAGGACGATGAAGAAGAAGCTGCCACTA<br>LH1788: CGCCTTACACCCGAAGTAGGTACCTCAT
AT1G31814, FRL2	LH1789: TACTTCCTGGTGTTGCTGTCCCATAT<br>LH1790: TAGAGAGCATAGACCTGTTCGAGCAG
AT3G12810, PIE1	LH1791: TGCTGCAGAGAACCGTTACAGGAATG<br>LH1792: GAGATTCGGATCATTTGGCCTAACCC
AT2G27100, SERRATE	LH1793: CGGATATAAACCCACCACCAATGCTG<br>LH1794: AGCATCTAGGTCTTGGTAGCTGCGCA
AT1G30970, SUF4	LH1795: ATTCTTATGCCTCTGGCCCAAACACT<br>LH1796: CTGCATTTATCGAGTTCATCTGGCTGG
AT4G26000, PEP	LH1799: TCTGGTGCATCAGTTCGTATTT<br>LH1800: CTCATCCTGTGCAGCATAAAAC
